# Development of Static Mixers for Millireactors and Their Production by Vat Photopolymerization

**DOI:** 10.3390/mi15060682

**Published:** 2024-05-23

**Authors:** Ivana Ćevid, Ivan Karlo Cingesar, Marijan-Pere Marković, Domagoj Vrsaljko

**Affiliations:** University of Zagreb Faculty of Chemical Engineering and Technology, Trg Marka Marulića 19, HR-10000 Zagreb, Croatia; icevid@fkit.unizg.hr (I.Ć.); icingesar@fkit.unizg.hr (I.K.C.); mmarkovi1@fkit.unizg.hr (M.-P.M.)

**Keywords:** additive manufacturing, vat photopolymerization, millireactor, static mixers, Fenton process

## Abstract

The addition of static mixers within reactors leads to higher productivity of a process and an additional increase in mass and energy transfer. In this study, we developed millireactors with static mixers using stereolithography, an additive manufacturing technology. Computational fluid dynamics (CFD) simulations were conducted to study the flow, identify potential dead volumes, and optimize the design of the millireactors. We produced five millireactors with various static mixers and one tubular reactor without static mixers, which served as a reference. The Fenton reaction was performed as a model reaction to evaluate the performance of the millireactors. We observed that some of the reactors with static mixers had air plugs that created a significant dead volume but still exhibited higher conversions compared to the reference reactor. Our results demonstrate the potential of stereolithography for producing intricate millireactors with static mixers, which can enhance the productivity of chemical processes.

## 1. Introduction

Additive manufacturing technology, while still in development, represents the future of manufacturing. Additive manufacturing technology will not change the fundamental structure of traditional production but will open up a wide range of production possibilities due to the relatively short time required to design and manufacture artifacts. In addition, it will enable the production of more geometrically complicated shapes and structures that were difficult or impossible to produce using classical methods.

With the development of materials suitable for additive manufacturing (AM), its scope is expanding. In the past, most AM technologies have used polymers as the manufacturing material. The use of metals for fabrication in AM processes has only increased in recent years with the development of new generations of systems for processing metal powders such as stainless steel, cobalt–chromium, gold, titanium alloys, and the like [1].

The quantity, size, and shape of the product and the desired quality of the same are the main parameters that determine the cost of any product produced by AM. The rule is that AM processes become less applicable as the size-to-product ratio increases. The simpler and less geometrically demanding a product is, the less need there is to use AM technologies, at least for now, until faster and cheaper AM technologies are developed. However, when it is necessary to produce geometrically complex and relatively small products, AM processes are an excellent choice for their production [2].

Vat photopolymerization is an AM process that creates objects by curing liquid photopolymer resins with a light source. There are several types of vat photopolymerization present on the market today, and one of them is stereolithography. Stereolithography (SLA) is a technology based on the process of photopolymerization in which the solidification of liquid resin is present. The liquid consists of photopolymers that harden under the electromagnetic radiation of a certain wavelength. The spectrum may include infrared light, the visible part of the spectrum, UV rays, X-rays, and γ-rays. The viscous resin used for stereolithography contains monomers and oligomers of non-cross-linked photopolymers and certain photoinitiators. Exposure of such photopolymers to electromagnetic radiation leads to a spontaneous polymerization process—liquid monomers and oligomers transform into solid polymers [2,3].

A crucial part of any chemical process is the chemical reactor, where reactants convert into products during the chemical reaction. Nowadays, the desire to be competitive in the market usually leads to the use of large-volume reactors to reduce production costs. Massive volumes lead to poorer energy and mass transfer, resulting in process selectivity problems and lower conversion. One of the possible solutions is the use of microreactor systems (millireactors and microreactors), in which reactants are brought into contact under optimal conditions, such as pressure and temperature. Excess heat can be supplied or removed from the microreactor system, which results in a process selectivity increase. Compared to microreactors, millireactors are increasingly used in industry due to the lower pressure drop in the system and higher pipe flow rate [4,5].

Batch reactors, which operate in batch or discontinuous mode, are conventional reactors. Compared to conventional reactors, microstructured reactors are much smaller. The internal parts of microstructured reactors are between a few millimeters and a few nanometers in size and are referred to as microstructured reactors or microreactors. They are classified according to the dimensions of the internal structural units into millireactors (1 mm to 10 mm), microreactors (100 nm to 1 mm), and nanoreactors (1 nm to 100 nm) [6,7].

The fundamental difference between microreactors and millireactors lies in their dimensions. It is important to mention that there are no significant differences between milli- and microreactors in terms of control methods, properties, and the outcome of the process. For these reasons, their characteristics can be observed and explained together; although, of course, the differences in characteristics are more obvious in comparison with conventional reactors.

Microreactors are characterized by small volumes with limited production, making them suitable for laboratory-scale optimization of flow chemistry reactions [8,9]. In contrast to microreactors, millireactors have wider channels (an order of a few millimeters), which results in a lower pressure drop and higher pipe flow rate. For these reasons, they are more suitable for industrial use. Due to their improved efficiency, capacity, and handling strength, millireactors are a middle choice between conventional reactors and microreactors [4].

The advantages of microreactors are excellent energy and mass transfer characteristics, low material usage, and low waste output. Their disadvantages are an extensive pressure drop, a tendency to clog the channel, and a low flow rate. The advantages of millireactors are a lower pressure drop, a high flow rate, and the ability to handle solids, while their disadvantage is poorer energy and mass transfer. As the size of the millireactor increases, the mixing efficiency decreases, which can be improved by the addition of mixing-promoting elements. In millireactors, it is common to experience channel plugging due to sediment formation due to a low-pressure drop in the system.

The idea of switching from batch to continuous processes is becoming more attractive in the industry, as they allow for economical production and the input of larger volumes of input streams. Continuous production reduces energy consumption and waste generation compared to the equivalent amount of product produced in batch processes. Since many of these processes rely on mixing and heat transfer, static mixers are increasingly being integrated into process systems. Process intensification is a goal of chemical engineering, which is to strive for processes with safer working conditions, lower waste production, lower energy consumption, and higher productivity. New applications and designs of heat exchangers, reactors, and static mixers are being explored that have several advantages over batch production and mechanical mixers. The main features that enable the use of these devices in various industries (chemical, pharmaceutical, food, polymer synthesis, paint and resin, water treatment, petrochemical, and others) are extremely small footprints, low operating and maintenance costs of the devices, short distribution residence times, improved selectivity due to enhanced mixing and isothermal operation, fewer by-products, and improved safety [10,11,12,13,14].

For economic and environmental reasons, it is very important to characterize mixing in industrial processes because mixing directly affects process efficiency and the number of byproducts released. Static mixers distribute the liquid flow through a series of fixed elements in directions transverse to the main flow. In this type of mixing, the mass transfer occurs by convection rather than diffusion [10,15,16,17].

The aim of this work is to develop reactor systems with static mixers and investigate the influence of static mixers on the reaction rate. The reaction used was Fenton oxidation of the organic pollutant—dye Reactive Blue 182. Stereolithography, an AM technology, was used to fabricate five millireactors. One fabricated millireactor is an ordinary tubular reactor that serves as a reference, and the other four millireactors have static mixers. The research will test the hypothesis that all elements of static mixers improve the transfer of matter and energy, resulting in a higher reaction rate.

## 2. Materials and Methods

### 2.1. Materials and Chemicals

Stereolithography, an additive manufacturing technology, was used to make the millireactors. The material used to make the millireactors is polyacrylate photopolymer resin. The resin version used is Clear V4 (FLGPCL04), manufactured by Formlabs Inc., Somerville, MA, USA.

The following chemicals were used in this work: Isopropanol p.a. (C_3_H_8_O), Gram-Mol d.o.o., Croatia; 30% hydrogen peroxide solution (H_2_O_2_), T.T.T. d.o.o., Sveta Nedelja, Croatia; ferrous sulfate heptahydrate (FeSO_4_ × 7H_2_O), Kemika d.d., Zagreb, Croatia; azo dye C.I. Reactive Blue 182 (Cibacron Blue F-R), abbreviation RB182, Ciba-Geigy Corp, Basel, Switzerland; 96% sulfuric acid solution (H_2_SO_4_), Lach-Ner s.r.o., Neratovice, Czech Republic; sodium hydroxide (NaOH), Lach-Ner s.r.o., Neratovice, Czech Republic; sodium sulfite (Na_2_SO_3_), Honeywell Riedel-de Haën AG, Seelze, Germany; and redistilled water.

### 2.2. Devices

Form 2 is a 3D printer from Formlabs Inc., Somerville, MA, USA, which is based on the SLA process. As mentioned earlier, this process uses a laser as a light source. The Form 2 printer emits light with a wavelength of 405 nm, which polymerizes acrylate monomers (in the form of a viscous liquid) into long polymer chains that are in a solid state.

After making the models on the SLA printer, they must be processed in a post-curing chamber equipped with a heater and an LED strip. The heater raises the temperature in the chamber to 60 °C, and the LED strip emits light with a wavelength of 405 nm. The millireactors were subjected to 90 min of post-curing in the chamber, during which additional cross-linking occurred, leading to an improvement in the mechanical properties of the material (increased tensile strength of the material) [18].

In this work, spectrophotometric analysis is used to determine the dye concentration in a sample by measuring the amount of light absorbed at a wavelength of 610 nm (maximum absorbance of the dye, shown in Figure S32). It is performed using a UV-1601 spectrophotometer (Shimadzu, Kyoto, Japan).

### 2.3. Millireactor Design and Construction

Computer-aided design (CAD) programs are used to design three-dimensional objects on a computer. The Autodesk Fusion 360 CAD program was used to design the five millireactors. One of the millireactors is a tubular millireactor, which serves as a default compared to the other four millireactors, which have built-in static mixers. Reactors with static mixers have the same number of static elements, 35 elements each.

Prior to the laboratory experiment, a computational fluid dynamics (CFD) simulation was performed. With the simulation results, it is possible to predict the fluid flow through the reactor. With this method, it is easier and faster to obtain valuable information about the reactor and to modify the geometry of the static mixer or the reactor itself. CFD saves money and time by allowing faster change, thus proving to be a valuable tool in engineering. For numerical analysis, Autodesk CFD 2021 software was used. The volume flow rate simulated for all reactors was 100 mm^3^ min^−1^.

Once the millireactor models are designed, they must be prepared for production in the PreForm program. The layer thickness used for all reactors is 0.025 mm (maximum resolution). All millireactors are oriented in a similar way. The goal of model orientation is a more successfully designed millireactor, where there is no residue of the resin and clogging of the channels during 3D printing. This is followed by processing in an additional curing chamber where the material is subsequently cured. The additional irradiation time of all millireactors with the 405 nm light in the curing chamber is 90 min.

## 3. Results and Discussion

Figure S1 in the Supplementary Materials shows the schematic of the tubular millireactor in the Fusion 360 program, showing the dimensions used for the construction of the millireactor tube. The diameter of the tube is 2 mm, and the height of the reactor is 8 mm.

**Figure 1:**
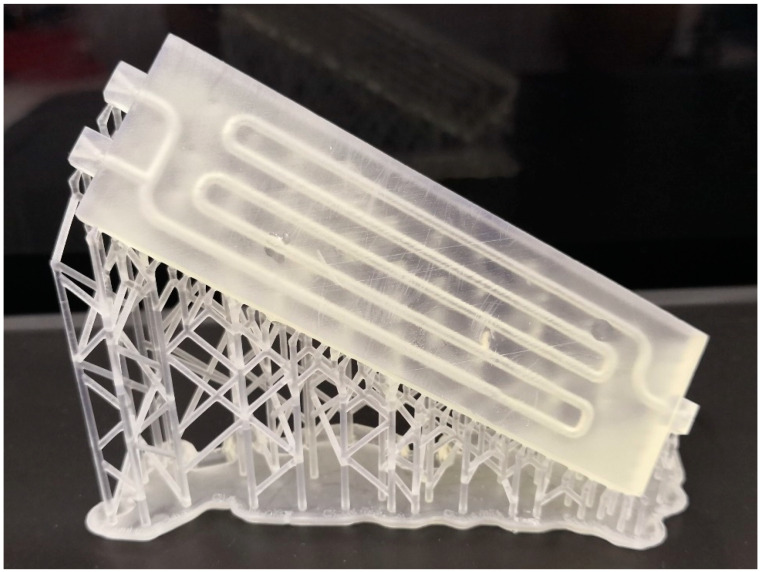
Tubular millireactor after fabrication on a Form 2 printer.

After the design, the millireactor was printed on an SLA printer (Formlabs Inc., Somerville, MA, USA). After that, the tubular millireactor must be cleaned of excess resin and cured in the chamber for post-curing, and the support structures must be removed. Figure 1 shows the manufactured tubular millireactor with the support structures.

**Figure 2:**
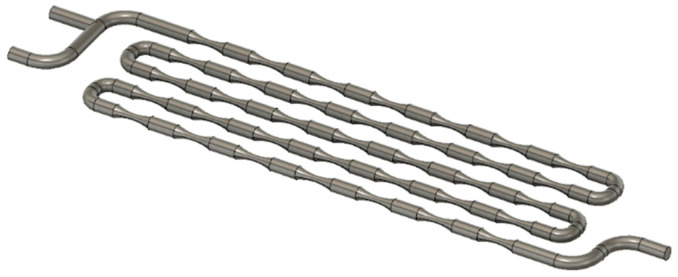
Venturi millireactor pipeline in Fusion 360.

The height and width of the Venturi millireactor are the same as the previous millireactor. The tube diameter is 2 mm, and the minimum constriction diameter of the static mixers is 1 mm. Figure 2 shows the piping of the Venturi millireactor.

After the construction of the Venturi millireactor (Figure S2 in the Supplementary Materials), the same treatment procedure is applied: cleaning off the excess resin with isopropanol and compressed air, followed by curing in the chamber for additional curing and removal of the support structures.

**Figure 3:**
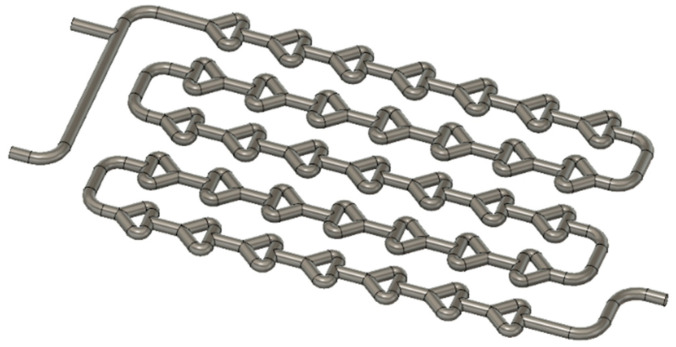
Chaos millireactor pipeline in Fusion 360.

The width of the Chaos millireactor is 60 mm, and the height is 8 mm. The diameter of the tube through the entire pipeline is 2 mm. In this design, the elements of the static mixers are achieved by branching one tube into two tubes, which are later joined to form one tube, as shown in Figure 3.

The fabricated Chaos millireactor after washing the excess photopolymer resin, processing it in the chamber for additional curing, and removing the support structures is shown in Figure S3 in the Supplementary Materials.

**Figure 4:**
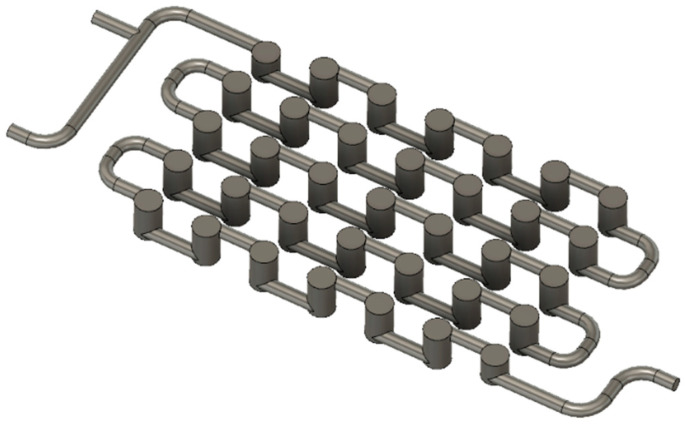
Illustration of pipelines with larger cylinders in the Cyclone millireactor in Fusion 360.

The height of the Cyclone millireactor is 12 mm, while its width is 46 mm. The diameter of the tube with a circular cross-section is 2 mm. Static mixers are designed so that there are smaller cylinders with a diameter of 1.5 mm inside the cylinders with a diameter of 4 mm (Figure 4), which have the same height (Figure S4 in the Supplementary Materials).

The inner cylinders with a diameter of 1.5 mm are solid, and the outer cylinders and piping with a diameter of 4 mm are hollow. As the fluid flows through the reactor, it is forced into static mixers and flows around smaller cylinders, reminiscent of the centrifugal motion of particles in a cyclone separator.

**Figure 5:**
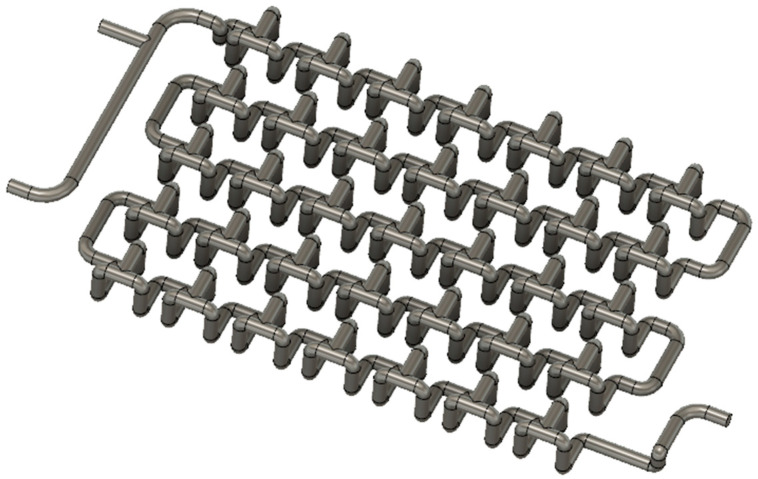
Mobius millireactor pipeline in Fusion 360.

The Mobius millireactor is 60 mm wide and 14 mm high. The diameter of the tube with a circular cross-section is 2 mm. One tube branches into two tubes, similar to the Chaos millireactor, except that in this design, tube branching also occurs in the third dimension (Figure 5).

The Fenton reaction for the decolorization of the dye Reactive Blue 182 (RB182) was used as a model reaction to analyze the flow rate and reaction rate. The concentrations of reactants to be used in the tubular reactors were determined by a batch reactor experiment—under optimal conditions, the reaction proceeds moderately slowly, and a color change from blue to relatively colorless is visible.

After the reaction products have left the millireactor, approximately 3 mL of the sample must be collected for spectrophotometric analysis. It is necessary to stop the reaction during sample collection in vials or tubes to determine the absorbance value of the dye at the exit of the reactor as accurately as possible.

Sodium sulfite (Na_2_SO_3_) was used as a quencher for the Fenton reaction (Equation (1)) [19,20].

Na_2_SO_3_ + H_2_O_2_ → Na_2_SO_4_ + H_2_O (1)

**Figure 6:**
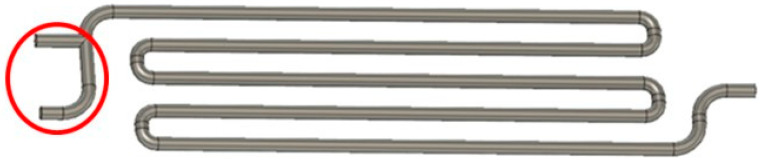
The part of the millireactor in which the reaction does not take place is circled red.

The total volume of the millireactors is read from the virtual model in Fusion 360. This volume is a theoretical volume. After the object is manufactured by additive manufacturing, there will certainly be some irregularities, so the actual volume will differ from the one given in the CAD program. The measured volumes of the millireactors were obtained by weighing empty millireactors with dry channels and millireactors filled with distilled water. The reaction volume of the millireactor, which is needed to be able to calculate the residence times of the reaction, was also calculated. The reaction volume is the volume in which the reaction takes place, i.e., in which the reactants are in contact with each other. Figure 6 shows the volume of the reactant inlet tube (highlighted in red), which was subtracted from the total volume of the millireactor to obtain the reaction volume. However, the reactant volume had to be corrected for the discrepancies between the theoretical and measured volumes. The final part to be included in the reaction volume is the volume of a 1 mm diameter polytetrafluoroethylene (PTFE) tube connected to the outlet of the millireactor. The same method was used for other millireactors.

**Figure 7:**
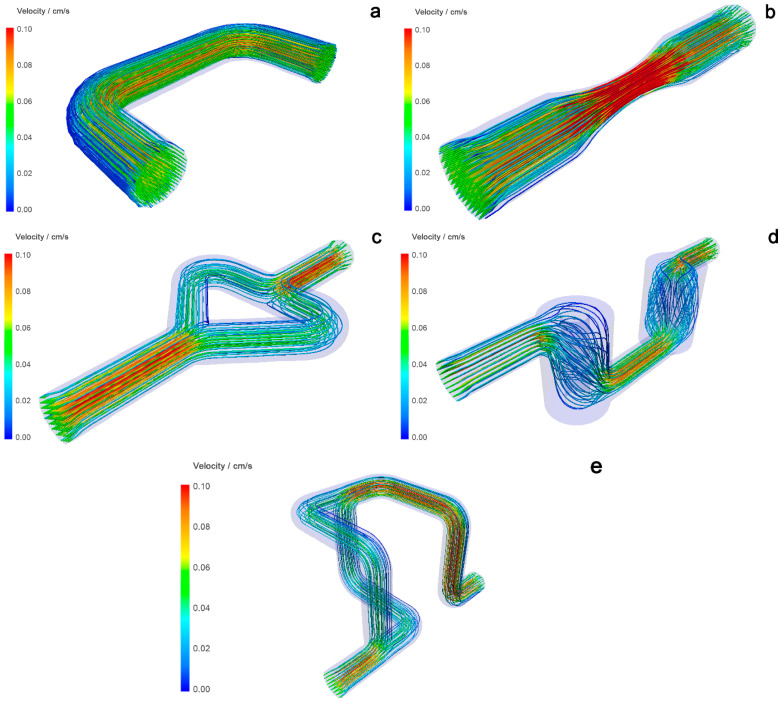
Velocity field tracers of the static mixers from Autodesk CFD 2021 software: (**a**)—tubular reactor, (**b**)—Venturi reactor; (**c**)—Chaos reactor; (**d**)—Cyclone reactor; (**e**)—Mobius reactor.

The CFD models of the reactors in Figure 7 show the velocity distribution in the ducts and indicate the formation of dead volumes in some reactors. The mixing efficiency in the reactors is estimated from the velocity change, which is shown in different colors. Static mixers that show a larger difference in velocity (change from blue to red and vice versa) are considered to have better mixing efficiency [21]. A tubular reactor (Figure 7a) shows a uniform distribution of velocity traces through the reaction volume, indicating poor mixing. The Venturi reactor (Figure 7b) shows better mixing than the tubular reactor because the velocities become higher as they pass through the narrower part of the tube and slow down as the cross-sectional area in the wider part of the tube becomes larger. The Chaos reactor (Figure 7c) shows a decrease in velocity as it reaches the branching part of the tube, indicating that the pressure in this part drops and the velocity increases thereafter, indicating good mixing. The Cyclone reactor (Figure 7d) shows a sharp drop in pressure and velocity in the cylindrical parts. This could mean that the mixing in each mixing element is good. However, the main problem with this static mixer is the presence of dead volume. This phenomenon is visible in the upper and lower parts of the cylinder in the simulation results, with an absence of velocity field tracers—close to blue tracers. The Mobius reactor (Figure 7e) shows large velocity differences in 3D branching parts. When the flow branches, the velocity and pressure in the mixing element decrease and then increase again. According to the simulations performed, both reactors with branching mixing elements, the Chaos and Mobius reactors, indicate the best potential for efficient mixing.

**Figure 8:**
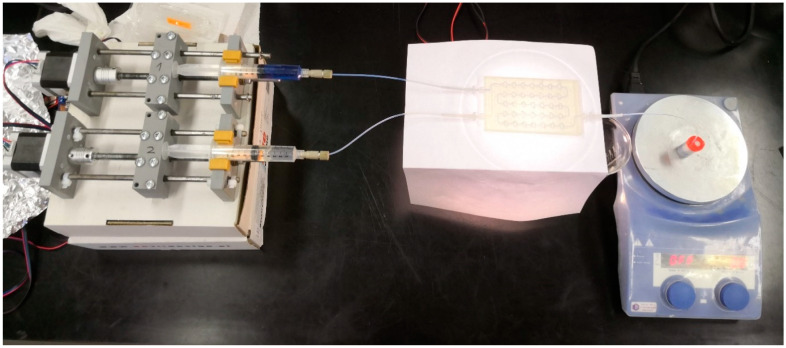
The reaction system consists of pumps, syringes, connectors, a reactor, and a sample vessel on a magnetic stirrer. In addition, a 1 mm diameter PTFE tube connected to the outlet of the millireactor is visible.

In this system (Figure 8), piston pumps controlled by a computer and two Luer Lock syringes (BD, Franklin Lakes, NJ, USA) containing reagents were used. Luer Lock syringes are connected to a 1 mm diameter PTFE tubing connected to the millireactor inlet using a Luer Lock adapter (Cole–Parmer, Vernon Hills, IL, USA) and a poly(ether ether ketone) (PEEK) connector (Cole–Parmer, Vernon Hills, IL, USA) around which Teflon tape is wrapped for better sealing with the adapter. PTFE tubing was connected to the inlet of the millireactor using a Cole–Parmer (Vernon Hills, IL, USA) reducer made of polypropylene. The other end of the reducer is connected to the inlet of the millireactor using a liquid resin and a laser emitting 405 nm light. With a smaller diameter end, the tube reducer is placed vertically at the inlet of the millireactor, and then a liquid resin is placed around the joint, which is cured with a laser. This type of connection ensures a good seal.

All Fenton reactions were performed in the same manner. Two Luer Lock syringes were placed on the pumps. One syringe contained an aqueous solution of hydrogen peroxide (*γ* = 81.6 mg L^−1^) and the other contained an aqueous solution of ferrous sulfate (*γ* = 0.6 mg L^−1^) and dye RB182 (*γ* = 120 mg L^−1^). For both aqueous solutions, the pH was adjusted to approximately 3.1. The desired flow is then set at the pump interface and a double residence time is waited to establish a steady state. The residence time is the time it takes for a reactor volume to run out. Samples for spectrophotometric analysis were collected at the outlet of the millireactor (i.e., at the end of the Teflon tubing connected to the millireactor, Figure 8). For flows greater than 500 μL min^−1^, samples were collected in 14-mL vials; for less than 500 μL min^−1^, samples were collected in capped containers.

The collection rate of samples for spectrophotometric analysis varied depending on the given flows, and, therefore, in order to stop the reaction, at lower flows (long duration of sample collection), it was necessary to add the reaction-stopping substance in rates. The quencher used, i.e., the reaction-stopping substance, was sodium sulfite. At flows of less than 60 μL min^−1^, sodium sulfite was added five times during sample collection. For flows of 500 μL min^−1^ to 60 μL min^−1^, sodium sulfite was added three times, and for flows greater than 500 μL min^−1^, it was added twice.

The reactions were performed in all millireactors with the same flows to determine how the flow rate affects the flow within the millireactor. Table 1 shows the individual flows at which the reactions were performed. The individual flow means that each pump has the standard flow in Table 1, so the total flow is their sum.

The Reynolds number values (Equation (2)) were also calculated using these flows. They can be used to determine whether the flow inside the millireactor is turbulent or laminar. Due to the complex geometries of the other millireactors with static mixers, the Reynolds number was only calculated for the tubular millireactor.

(2) $$Re=\frac{vd\;\rho }{\eta }$$

In Equation (2), *v* represents the fluid flow velocity, *d* represents the linear characteristic of the observed system (in this case, the diameter of the circular cross-section tube), *ρ* represents the density of the fluid, and *η* represents the dynamic viscosity of the fluid. In the preparation of the dye solutions, 30 mg of RB182 dye in 250 mL of distilled water was used. Such small amounts of dissolved dye are considered to have a negligible effect on the density and viscosity of water, so in the calculation for density and viscosity, values of the density and viscosity of water at the reaction temperature (25 °C) are taken. The density of water at a temperature of 25 °C is 997.044 kg m^−3^, and the dynamic viscosity is 0.8891 mPa s [22,23].

The results of the Fenton reactions performed are spectrophotometric values (absorbance values), from which the conversion of the reaction must be determined. The conversions are calculated using molar fluxes according to Equation (3) as follows:

(3) $$X=\frac{\dot{n_{1\;}}-\;\dot{n_{2}}}{\dot{n_{1}}}$$

where *ṅ*_1_ is the molar flux of the initial solution and *ṅ*_2_ is the molar flux of the final solution, i.e., the sample to be analyzed. From the absorbance values, using the calibration chart, the mass concentration of the dye in the sample is calculated, and the molar fluxes are calculated according to Equations (4) and (5) as follows:

(4) $$\dot{n_{1}}=\frac{\gamma_{1}}{M_{r}}\;\times \;Q_{1}$$

(5) $$\dot{n_{2}}=\frac{\gamma_{2}}{M_{r}}\;\times \;Q_{\mathrm{uk}}$$

where *γ*_1_ is the mass concentration of the initial solution, *γ*_2_ is the mass concentration of the final sample, *Q*_1_ is the flow rate of the aqueous solution with dye, and *Q*_uk_ is the sum of the flow rates of both pumps.

In addition to the Fenton reactions in millireactors with the same flow rates, Fenton reactions in millireactors with fixed residence times were also performed. Fixed residence times were determined, and the corresponding flux for each millireactor with respect to its volume was calculated using Equation (6).

(6) $$\tau =\frac{V}{Q}$$

where *τ* is the residence time or residence time in the reactor.

Table 2 shows the specified residence times and the corresponding flows of the individual millireactors to achieve them. The indicated flows are individual, i.e., the same are set for both pumps.

During the Fenton reactions, it necessary to wait for two residence times (to establish a steady state) before samples could be taken for spectrophotometric analysis. A quencher (Na_2_SO_3_) was added to the samples during sample collection. Considering the flow rate used, Na_2_SO_3_ was added before sample collection, after sample collection, and, if necessary, during sample collection. After taking about 3 mL of the sample, two drops of sulfuric acid were added to dissolve the Fe(OH)_3_ flakes. The sample was then filtered and taken for spectrophotometric analysis within about 15 min to avoid major deviations from the true absorbance values at the millireactor outlet. Sakalis et al. used a polytetrafluoroethylene (PTFE) filter to filter azo dyes with sulfone groups, and it was, therefore, selected for this test [24].

When the reactants (aqueous dye solution and peroxide) come into contact with the millireactors, the dye solution is diluted and, therefore, the conversions should be calculated using the molar fluxes rather than the absorbance directly.

**Figure 9:**
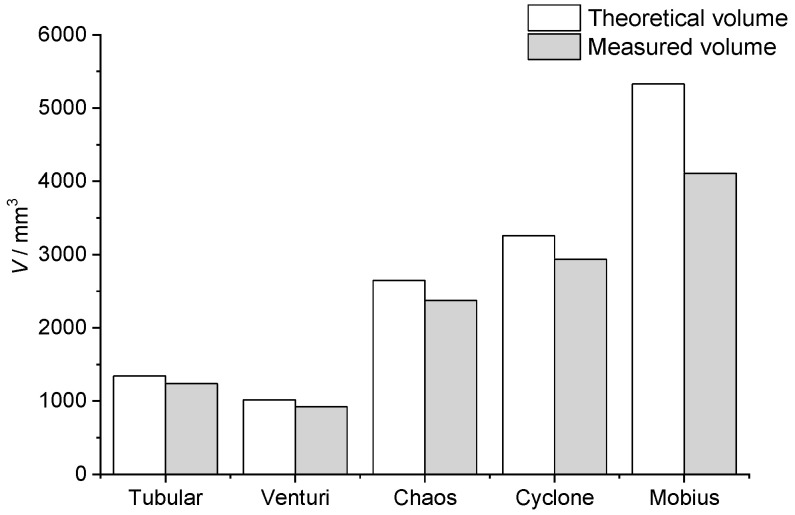
Graphical representation of the differences between theoretical and measured volumes for all millireactors.

Table 3 shows the total theoretical volume of the millireactor (*V*_t.t._) read from the CAD program, the total measured volume of the millireactor (*V*_t.m._) determined by weighing the dry and full millireactor, the deviation of the theoretical from the measured volume (Δ*X*), the theoretical volume of the reactants path (*V*_p.t._) read from the CAD program, the path volume of the reactants with corrected value with respect to the deviation of the total theoretical and measured volume (*V*_p.c._), the reaction volume in which the total measured volume of the millireactor is summed with the path volume of the reactants with corrected value (*V*_r._), and the reaction volume to which the volume of the Teflon tube from the outlet of the millireactor (*V*_r.+o._) is summed. In Table 3 and Figure 9, it can be seen that all the manufactured millireactors have a smaller volume than the one predicted by the CAD model. The largest deviation of the measured volume from the theoretical one has the Mobius millireactor with 22.87%. A possible explanation lies in the values of the millireactor volume. Since the volume of the Mobius millireactor is the largest, there is a high probability that during production on the printer (which took the longest), there was subsequent polymerization of the channel due to the dispersion of the beam emitted by the laser. When cleaning the millireactors with isopropanol and rinsing with compressed air, not all channels can be completely cleaned due to the geometry of the Mobius millireactor. If the excess resin is not completely removed from them after they are placed in the post-curing chamber, the resin will polymerize and further fill the tubes, which could be an additional explanation for the relatively large deviation. This theory is supported by the fact that the next largest deviation belongs to the Chaos millireactor, whose geometry is very similar to that of Mobius. In the Chaos millireactor, one tube branches into two and then reconnects. The difference is that in Mobius, the tubes branch in the third dimension.

The experimental tube radius was calculated because it was smaller than the one predicted by the CAD model. Thus, to determine the experimental value of the Reynolds number, we also need an experimental radius, which is calculated to be 0.9618 mm.

Table 4 shows the calculated theoretical and experimental values of flow rates and Reynolds number. The Reynolds number is the dimensionless ratio of inertial force and frictional force. It is used as a criterion for predicting whether laminar or turbulent flow will occur. Laminar flow is present at Reynolds number values of less than 2300, after which unsteady flow occurs, and turbulent flow occurs at Reynolds numbers greater than 4000 [25,26,27].

In Table 4, it can be seen that all values of Reynolds numbers indicate laminar flows for all flows. Based on the values of flow velocities and Reynolds numbers, it can be observed that with a decrease in the radius (*r*_exp_. = 0.9618 mm, *r*_theor._ = 1.000 mm), there is an increase in flow velocities and Reynolds numbers.

**Figure 10:**
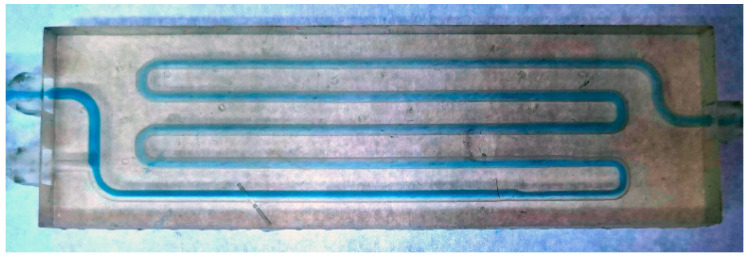
Tubular millireactor at a flow rate of 100 μL min^−1^.

**Figure 11:**
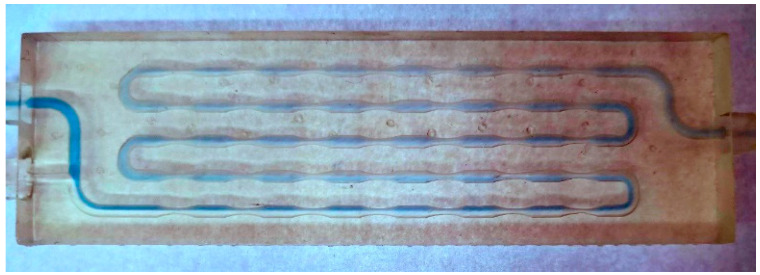
Venturi millireactor at a flow rate of 100 μL min^−1^.

**Figure 12:**
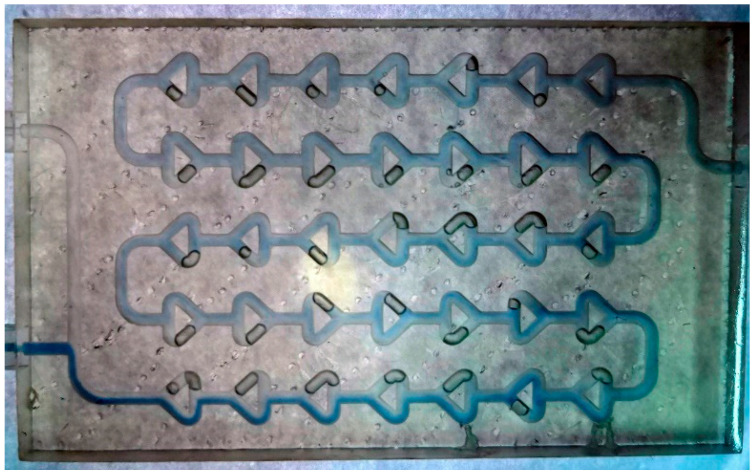
Chaos millireactor at a flow rate of 100 μL min^−1^. Residual air plugs, which create dead volume where the reaction does not take place, can be observed.

**Figure 13:**
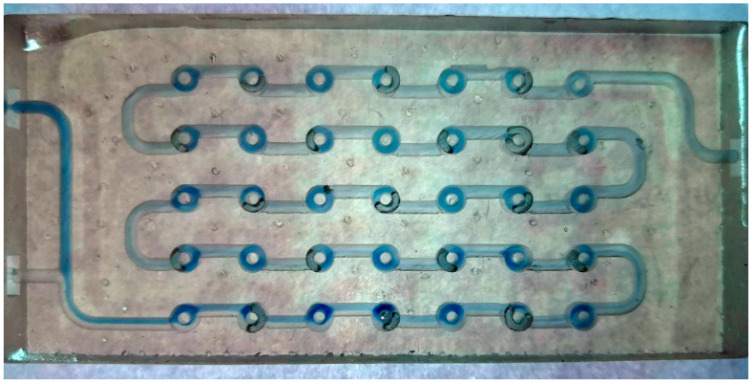
Cyclone millireactor at a flow rate of 100 μL min^−1^.

**Figure 14:**
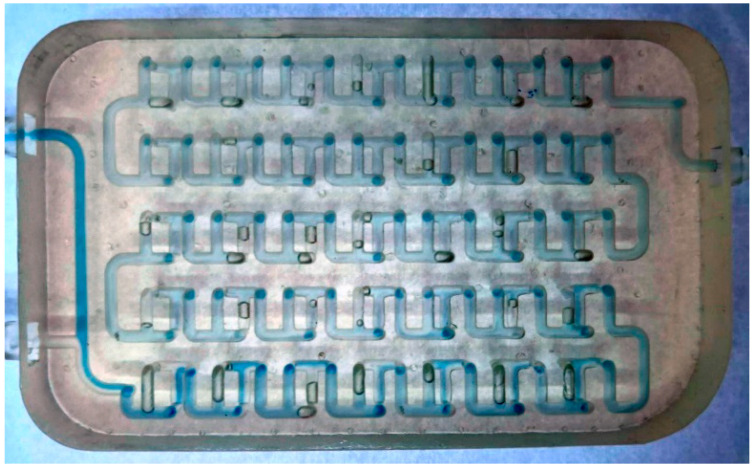
Mobius millireactor at a flow rate of 100 μL min^−1^. Residual air plugs, which create dead volume where the reaction does not take place, can be observed.

Figure 10, Figure 11, Figure 12, Figure 13 and Figure 14 show the flows inside all millireactors during the Fenton reaction at a flow rate of 100 μL min^−1^. Figures S6–S21 in the Supplementary Materials include images at higher flow rates, showing more violent mixing of reactant flows and leading to the conclusion that unsteady flow occurred, but it is clear from the calculated values of the Reynolds number that only laminar flows are possible in these millireactors. Thus, this phenomenon is attributed exclusively to mixing at the contact point of the reactants, where a sudden collision of the reactants occurs. In Figure 10, the line of the two reactant flows can be clearly seen, from which it is easy to conclude that laminar flow occurs at a flow rate of 100 μL min^−1^. It can also be seen that the color of the mixture is more homogeneous after the first knee drop. Flow rates below 4000 μL min^−1^ show similar behaviors to a flow rate of 100 μL min^−1^.

In the Chaos and Mobius millireactors, residual air plugs are observed after individual forks in parts of the pipeline, representing dead volumes where the reaction does not occur and further reducing the reaction volume. This reduction in reaction volume was not accounted for in the definition of reactor volume and represents a relatively significant experimental error due to which the measured conversions at certain residence times are lower than the actual ones.

We tried to get the bubbles out by tilting the reactors at all possible angles and increasing the flow of water and various organic solvents (acetone, isopropanol, ethanol). Even when we applied a very high liquid pressure, a small amount of air bubbles always remained inside. Although the air bubbles always remained inside because the amount was similar, the results were reproducible.

**Figure 15:**
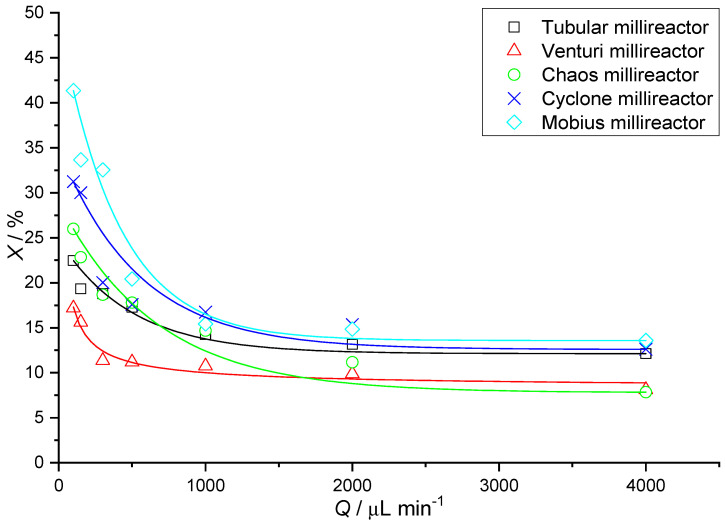
Graphical representation of the conversion values obtained for the millireactors at the same flow rates.

In Table 5, we notice a growing trend in the value of conversions with a decrease in the value of the flow rate used. This is expected because, at lower flow rates, the reactants remain longer in the millireactor; therefore, diffusion plays a greater role, resulting in better transfer of the substances. In Figure 15, it can be seen that the highest reaction yield values at the lowest flow rate (100 μL min^−1^) are obtained by the Mobius millireactor, followed by Cyclone and then Chaos. This can be explained by the values for the volume of the millireactor. Returning to Table 3, we can see that the downward trend of conversions in Figure 15 also monitors the decreasing values of the millireactor volume. The residence time is directly proportional to the volume of the millireactor. It can be concluded that a larger volume leads to a longer residence time and thus to higher conversion values.

**Figure 16:**
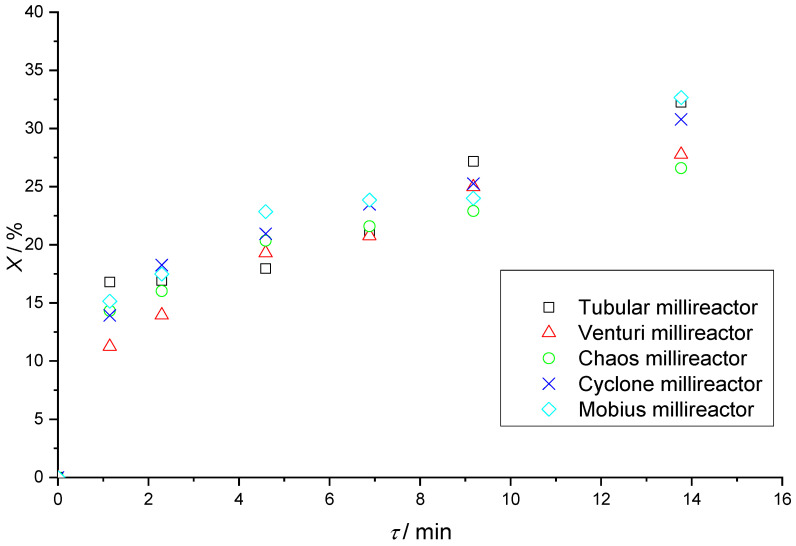
Graphical representation of the dependence of the conversions of all five millireactors on fixed residence times.

Table 6 shows the results of Fenton reactions in all five millireactors with fixed residence times. There is an increasing trend in the value of conversions with increasing residence time in the reactors, which is expected since the Fenton reaction is a relatively slow reaction under these initial conditions, achieving higher yields with longer reaction times. Figure 16 shows that the Mobius millireactor, the tubular millireactor, and the Cyclone, in that order, have the highest conversions.

At slower flows, the boundary between the two phases is clearly visible, and at faster flows, there is turbulent mixing at the beginning of the reaction volume caused by higher flow velocities. It should be noted that during the reactions in the Chaos and Mobius millireactors, the full potential of the static mixers was never realized because air plugs were retained in parts of the channel, as can be seen in Figure 12 and Figure 14. These air plugs and the dead volume force the fluid flow through the remaining volume of the reactor. Since the remaining volume of the reactor is much smaller compared to the full volume, the real residence time is much shorter than the calculated theoretical one used to create Figure 16. Although air plugs create a significant dead volume in the Chaos and Mobius millireactors, together with the simple tubular reactor, which has no dead volume and whose volume is fully utilized, they are still the reactors with the highest conversions. Since these reactors with effectively much shorter residence time have the same efficiency as the ones without dead volume, they prove the efficiency of these static mixers. Further research is needed to remove the air plugs and further increase the efficiency of the reactors. 

The Venturi millireactor has no true dead volume and its volume is fully utilized. Based on the results from CFD, we can see that most of the reactants flow very fast through the center of the channel and thus have a short residence time, i.e., much shorter than the average compounds. On the other hand, reactants passing close to the channel walls flow much slower, especially in the wider parts of the channel, creating a semi-dead volume in which the reactants are extremely slow. The situation is similar in the Cyclone reactor. The distribution of velocities in the Cyclone reactor with cylindrical static mixers indicates the existence of zones where the liquid moves extremely slowly, so we do not expect mixing there.

In addition to the presence of plugs, there are several reasons that can affect the conversion in a particular reactor. The reactors have different volumes, so the velocities are different, which affects the type of flow and the occurrence of turbulence. The different flow velocities depend on the design of the channels and static mixers, which further emphasizes the complexity of optimizing the design of static mixers.

## 4. Conclusions

In this work, five millireactors were designed and constructed using stereolithography. One of the millireactors is a simple tubular millireactor, and four millireactors have static mixers. 

When the measured volume of the millireactor was determined, the Mobius millireactor was found to have the largest deviation of 22.87% from the dimensions of the specified CAD model. Since it also has the largest volume, resulting in the longest production time on a 3D printer, it was concluded that the subsequent polymerization on the channel walls was most likely due to the scattering of the beam emitted by the laser. Another possible reason for the significant deviations is due to the cleaning of the millireactor channels themselves. If the excess resin is not completely removed, there will be a reduction in the tube diameter during subsequent curing, resulting in reduced volume after fabrication.

The values of the Reynolds numbers for the tubular millireactor at the total flow rates show that the expected flows are laminar at all flow rates. It was found that at higher flow rates in all millireactors, mixing occurs at the point of collision of the reactants, which could be visually interpreted as the phenomenon of unsteady flow. Considering the low values of the Reynolds number, it was concluded that this phenomenon is caused by higher flow rates, which cause a sudden mixing of reactants at the point of contact. At lower flow values, a typical laminar flow occurs in which two fluid streams are clearly visible. After the first bend, better mixing occurs, so this line is no longer so clearly visible. The same observations were made for reactions with fixed residence times. The obtained conversions of the millireactors at the same flow rates showed that the Mobius millireactor achieves the highest conversion, which is directly proportional to its volume. The larger the volume of the millireactor, the longer the residence time, resulting in higher conversion.

CFD simulations predicted that the Mobius and Chaos reactors would give the best results. Optimal conditions were considered in the simulations, which means that the presence of dead volume is minimal and can be observed, especially in the Cyclone reactor. The experimental results of the Fenton reaction at fixed residence times showed an increasing trend of conversions with increasing residence time. The Mobius millireactor, the tubular millireactor, and the Cyclone reactor showed the highest values. Air plugs were observed in the Mobius and Chaos reactors. They represent a dead volume in the reactor that led to incorrect calculation of residence times, as this could not be well quantified. The air plugs and the dead volume force the fluid flow through the remaining volume of the reactor. The remaining volume of the reactor is much smaller compared to the full volume, and the real residence time is much shorter than the theoretical one. Although the air plugs in these reactors create a considerable dead volume, these are still the reactors with the highest conversions, along with the simple tubular reactor, which has no dead volume and whose volume is fully utilized. This proves the efficiency of these static mixers; although, further research is needed to remove the air plugs and further increase the efficiency of the reactors. Also, this research demonstrates the efficiency of vat photopolymerization for the production of intricate millireactors with static mixers. 

## Figures and Tables

**Table 1:** The flows of the two syringes used to perform the reactions.

*Q*/μL min^−1^
4000
2000
1000
500
300
150
100

**Table 2:** Required flows in relation to the fixed residence times.

*τ*/min	*Q*/μL min^−1^				
	Tubular	Venturi	Chaos	Cyclone	Mobius
1.15	587	448	1059	1312	1823
2.29	294	224	529	656	911
4.59	147	112	265	328	456
6.88	98	75	176	219	304
9.18	73	56	132	164	228
13.77	49	37	88	109	152

**Table 3:** Display of the calculated volumes for the determination of the reaction volume.

Millireactor	*V*_t.t./_mm^3^	*V*_t.m./_mm^3^	Δ*X*/%	*V*_p.t./_mm^3^	*V*_p.c./_mm^3^	*V*_r._/mm^3^	*V*_r.+o./_mm^3^
Mobius	5328.89	4110.17	22.87	131.50	101.43	4008.74	4181.53
Cyclone	3258.45	2935.55	9.91	109.51	98.66	2836.89	3009.67
Chaos	2648.16	2374.29	10.34	131.50	117.90	2256.39	2429.18
Venturi	1018.67	921.05	9.58	71.81	64.93	856.12	1028.91
Tubular	1341.20	1240.77	7.49	71.81	66.44	1174.33	1347.12

**Table 4:** Theoretical and experimental values of flow velocities and Reynolds numbers.

*Q* (uk.)/μL min^−1^	*v* (theor.)/m s^−1^	*Re* (theor.)	*v* (exp.)/m s^−1^	*Re* (exp.)
8000	0.0424	95.19	0.0459	98.97
4000	0.0212	47.59	0.0229	49.48
2000	0.0106	23.80	0.0115	24.74
1000	0.0053	11.90	0.0057	12.37
600	0.0032	7.14	0.0034	7.42

**Table 5:** Determined conversion values of all millireactors at the same flow rates.

*Q*_1_/μL min^−1^	*X/*%				
	Tubular	Venturi	Chaos	Cyclone	Mobius
4000	12.12	8.12	7.84	12.61	13.58
2000	13.13	9.87	11.17	15.36	14.89
1000	14.19	10.74	14.68	16.74	15.42
500	17.38	11.16	17.79	17.59	20.42
300	18.47	11.83	18.67	20.03	32.55
150	19.32	15.62	22.83	30.01	33.67
100	22.47	17.18	25.98	31.24	41.35

**Table 6:** Conversion values of all reactors in relation to fixed residence times.

*τ*/min	*X*/%				
	Tubular	Venturi	Chaos	Cyclone	Mobius
1.15	16.79	11.26	14.33	13.92	15.15
2.29	16.89	13.96	16.02	18.26	17.49
4.59	17.97	19.30	20.35	20.95	22.85
6.88	21.18	20.76	21.60	23.48	23.85
9.18	27.18	24.98	22.91	25.26	24.00
13.77	32.25	27.77	26.60	30.77	32.90

## Data Availability

The original contributions presented in the study are included in the article, further inquiries can be directed to the corresponding authors.

## Supplementary Materials

The following supporting information can be downloaded at https://www.mdpi.com/article/10.3390/mi15060682/s1, Figure S1: Tubular millireactor scheme in Autodesk Fusion 360 software; Figure S2: Venturi millireactor after the construction; Figure S3: Manufactured Chaos millireactor.
