# Supplementary material for: Development of Static Mixers for Millireactors and Their Production by Vat Photopolymerization
Source: Micromachines (Basel). 2024 May 23;15(6):682. doi: 10.3390/mi15060682 (PMC11205379; doi:10.3390/mi15060682)
Supplement: Supplementary file 1 [file micromachines-15-00682-s001.zip › micromachines-2997472-supplementary.pdf]

Development of static mixers for millireactors and their production by vat photopolymerization

Ivana Ćevid*, Ivan Karlo Cingesar, Marijan-Pere Marković, Domagoj Vrsaljko*

University of Zagreb Faculty of Chemical Engineering and Technology,
Trg Marka Marulića 19, HR-10000 Zagreb, Croatia

icevid@fkit.unizg.hr, icingesar@fkit.unizg.hr, mmarkovi1@fkit.unizg.hr, *dvrsal@fkit.unizg.hr

ABSTRACT

The addition of static mixers within reactors leads to higher productivity of a process and an additional increase in mass and energy transfer.

In this study, we developed millireactors with static mixers using stereolithography, an additive manufacturing technology. Computational fluid dynamics (CFD) simulations were conducted to study the flow, identify potential dead volumes and to optimize the design of the millireactors. We produced five millireactors with various static mixers and one tubular reactor without static mixers which served as a reference. The Fenton reaction was performed as a model reaction to evaluate the performance of the millireactors. We observed that some of the reactors with static mixers had air plugs that created a significant dead volume, but still exhibited higher conversions compared to the reference reactor. Our results demonstrate the potential of stereolithography for producing intricate millireactors with static mixers, which can enhance the productivity of chemical processes.

Keywords: additive manufacturing, vat photopolymerization, millireactor, static mixers, Fenton process

SUPPLEMENT

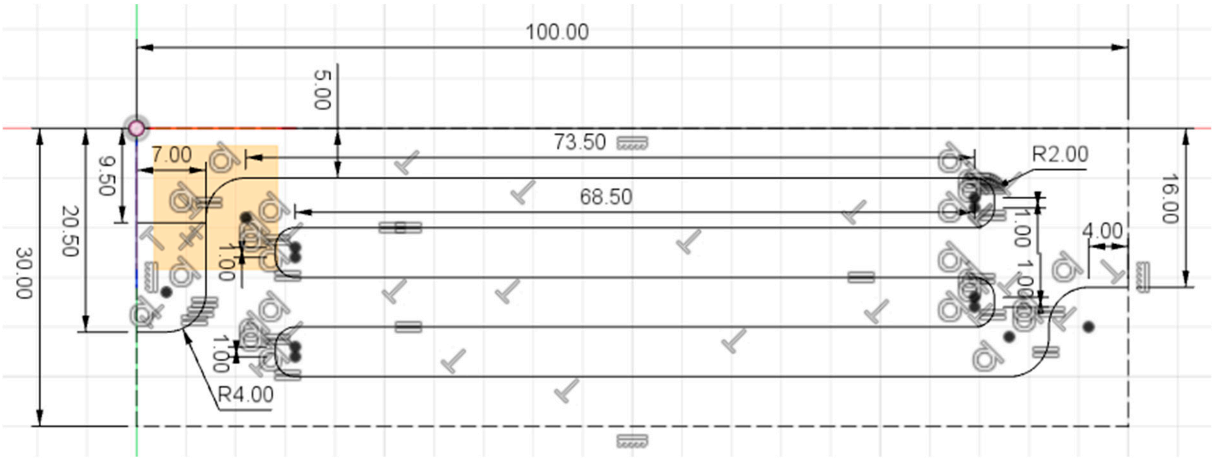

Supplementary Figure S1. Tubular millireactor scheme in Autodesk Fusion 360 software

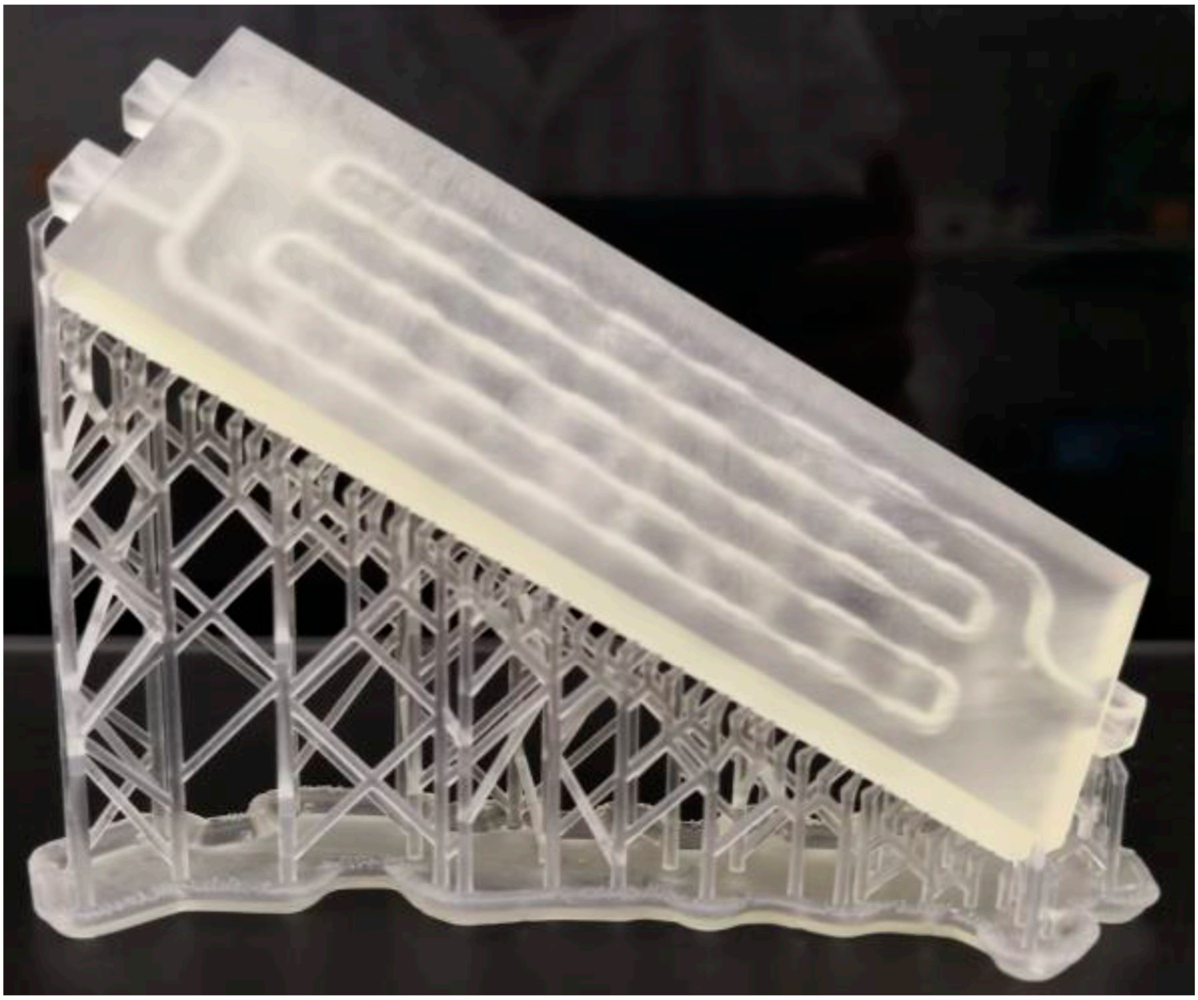

Supplementary Figure S2. Venturi millireactor after the construction

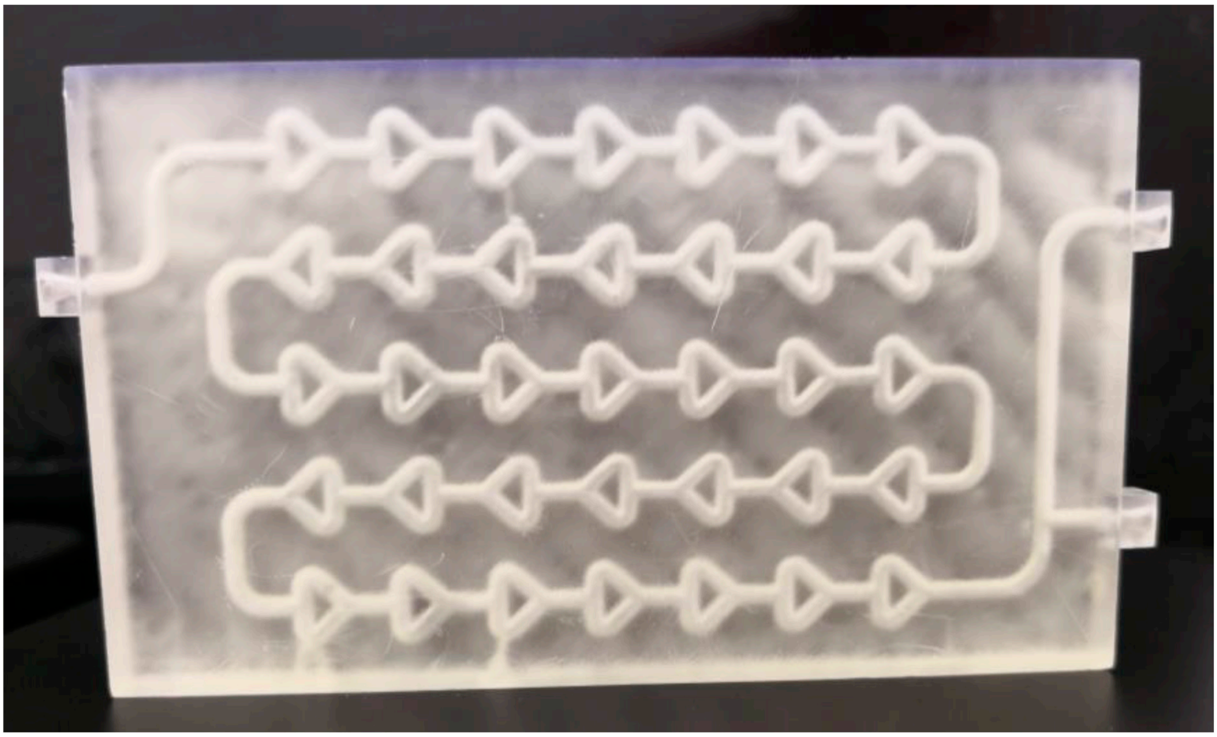

Supplementary Figure S3. Manufactured Chaos millireactor

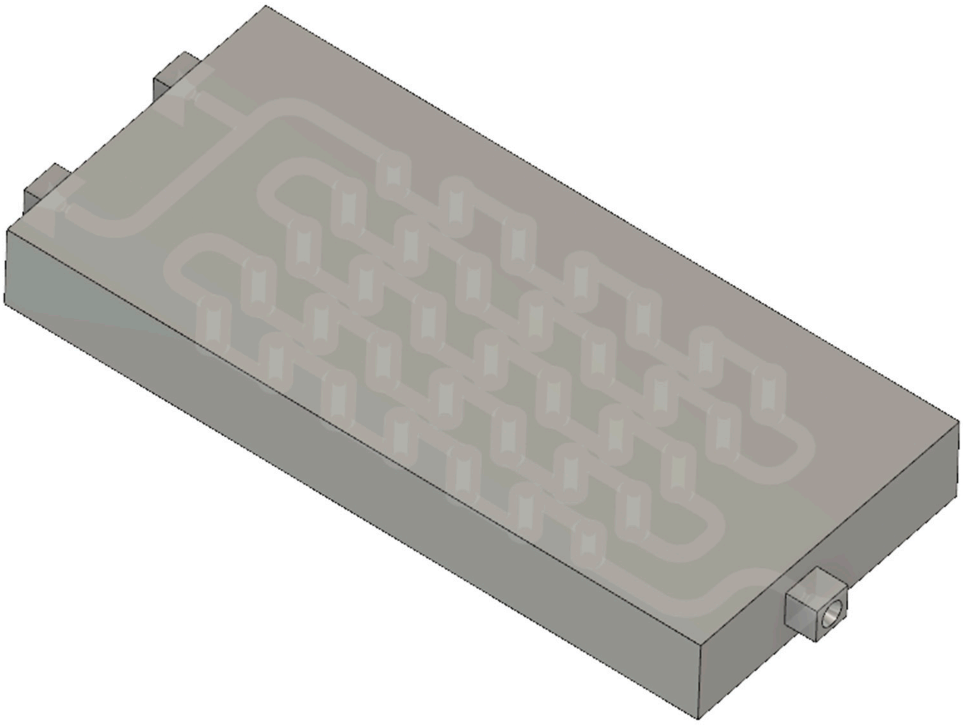

Supplementary Figure S4. Cyclone millireactor in Autodesk Fusion 360 software with visible internal cylinders

Calibration diagram

Supplementary Figure 5. shows a calibration diagram of the dependence of the absorbance of dye RB182 on mass concentration. It was used to calculate unknown sample concentrations required for further calculations of millireactor conversion at specific flow rates.

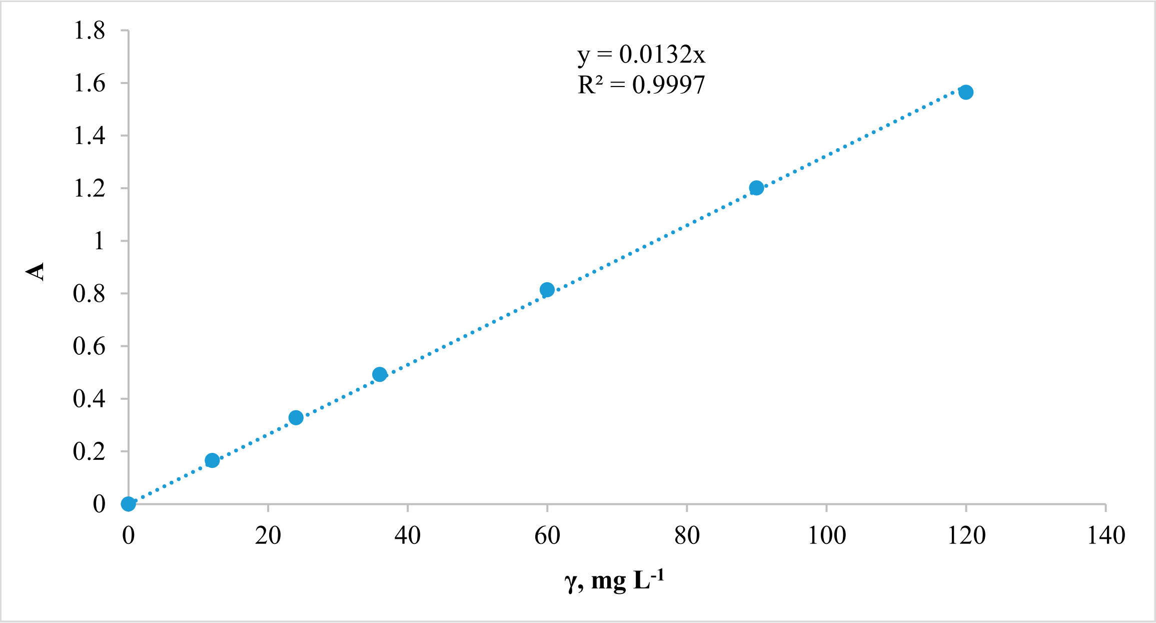

Supplementary Figure S5. Calibration diagram of dye RB182

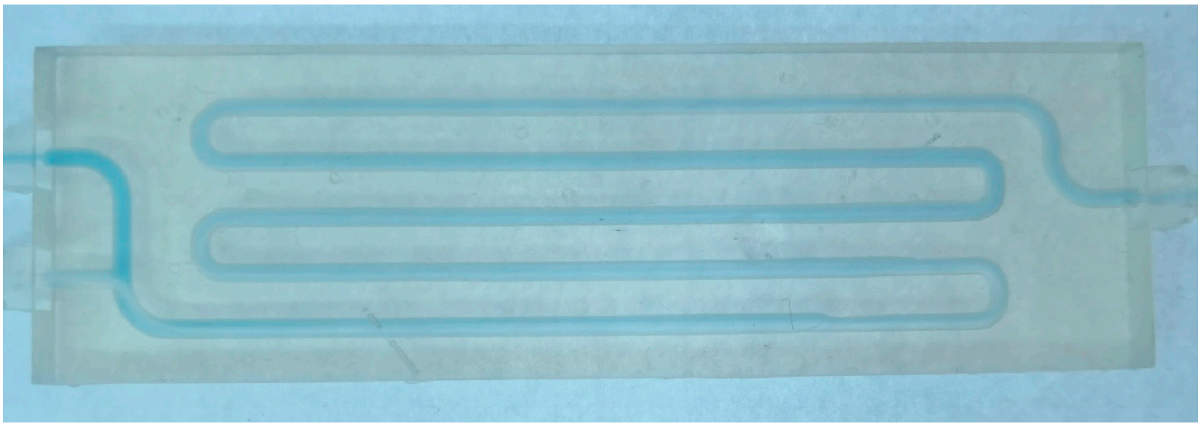

Supplementary Figure S6. Tubular millireactor at flow of 4000 μL min^{-1}

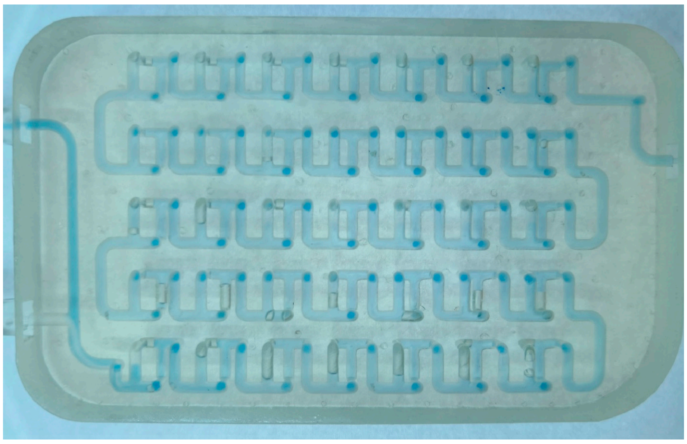

Supplementary Figure S7. Mobius millireactor at flow of 4000 μL min^{-1}

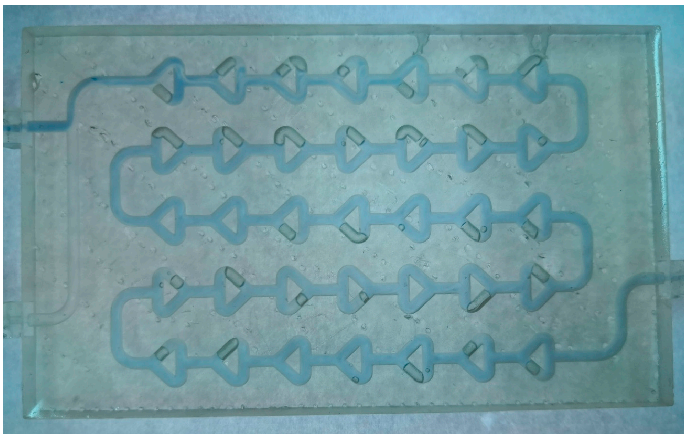

Supplementary Figure S8. Chaos millireactor at flow of 4000 μL min^{-1}

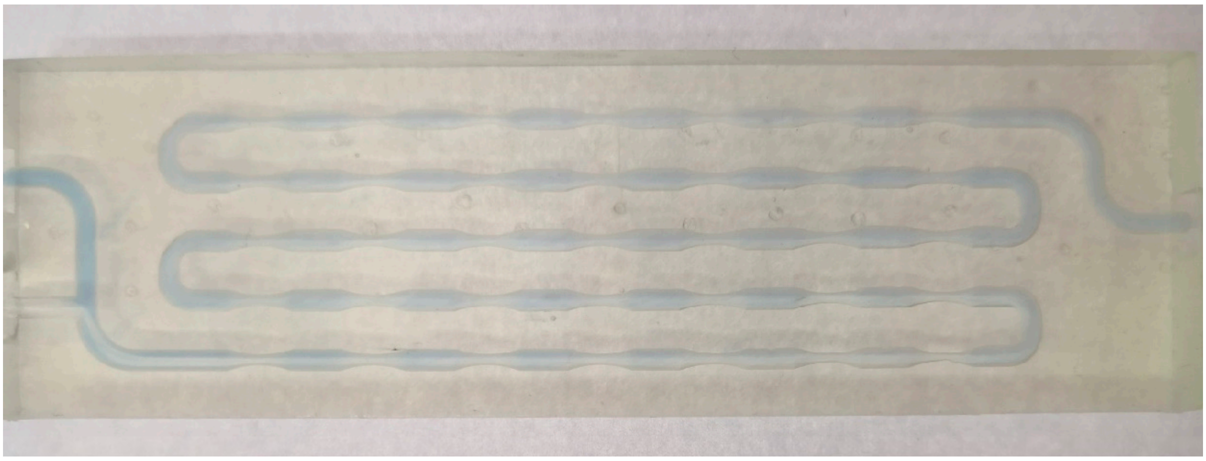

Supplementary Figure S9. Venturi millireactor at flow of 4000 μL min^{-1}

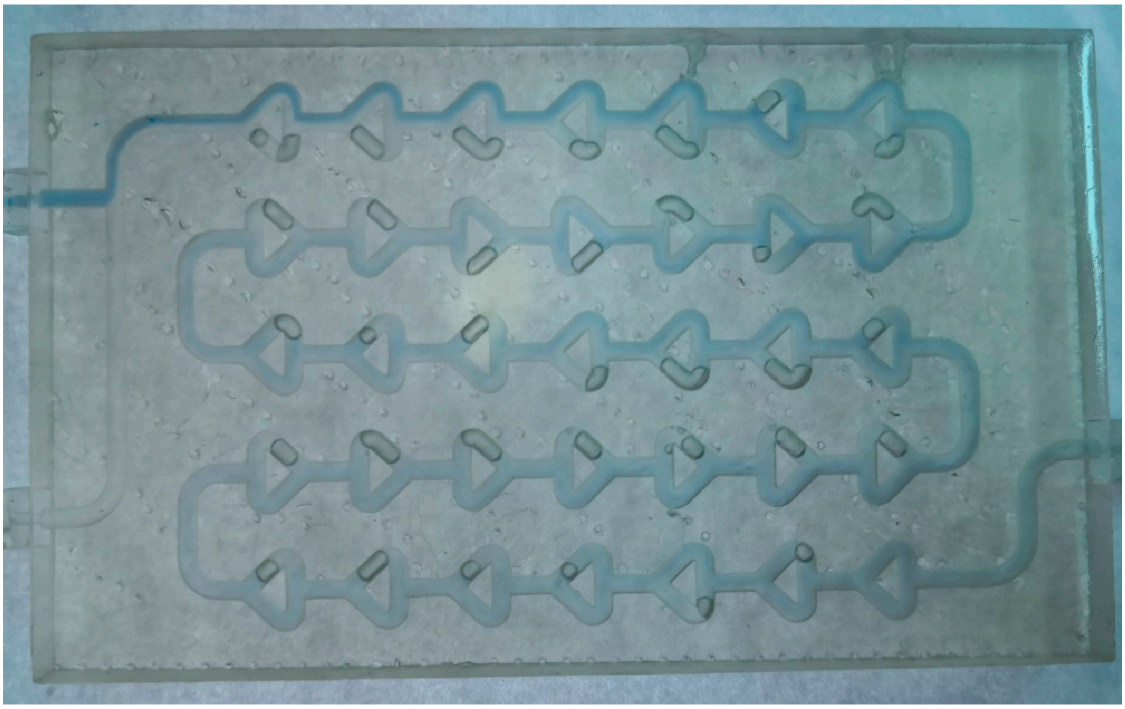

Supplementary Figure S10. Chaos millireactor at flow of 100 µL min^{-1}

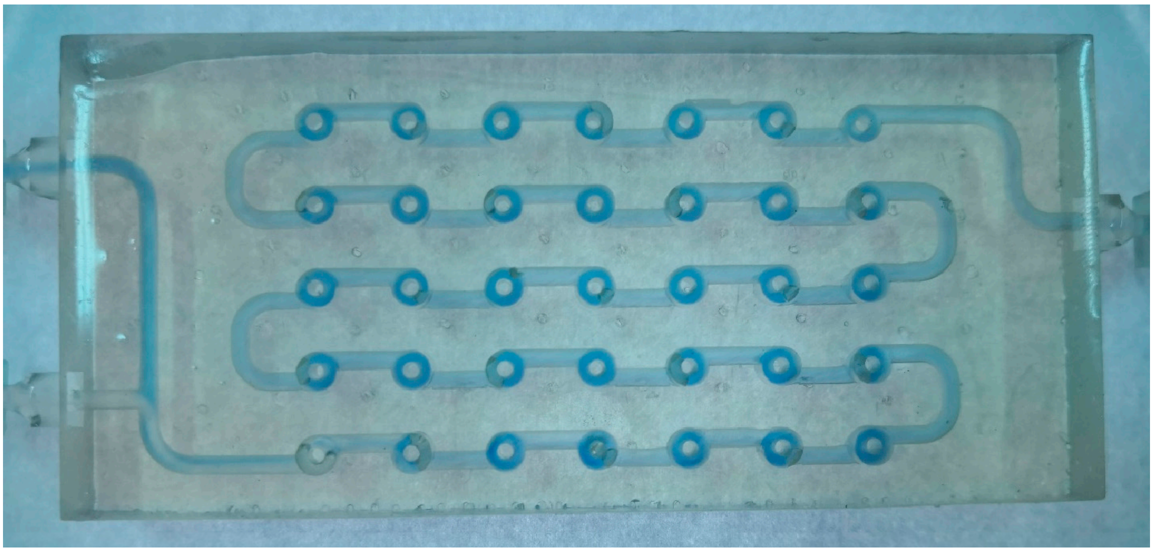

Supplementary Figure S11. Cyclone millireactor at flow of 4000 µL min^{-1}

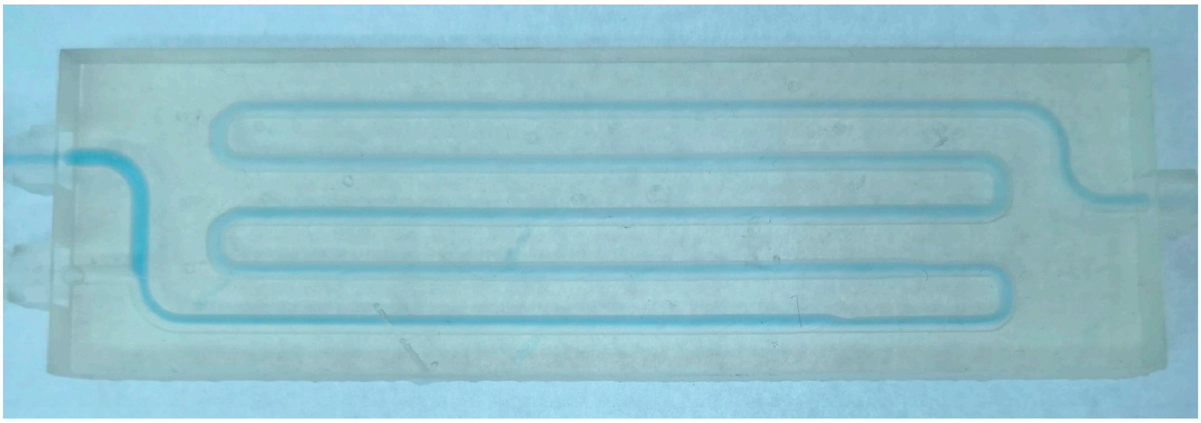

Supplementary Figure S12. Tubular millireactor at flow of 587 μL min^{-1}

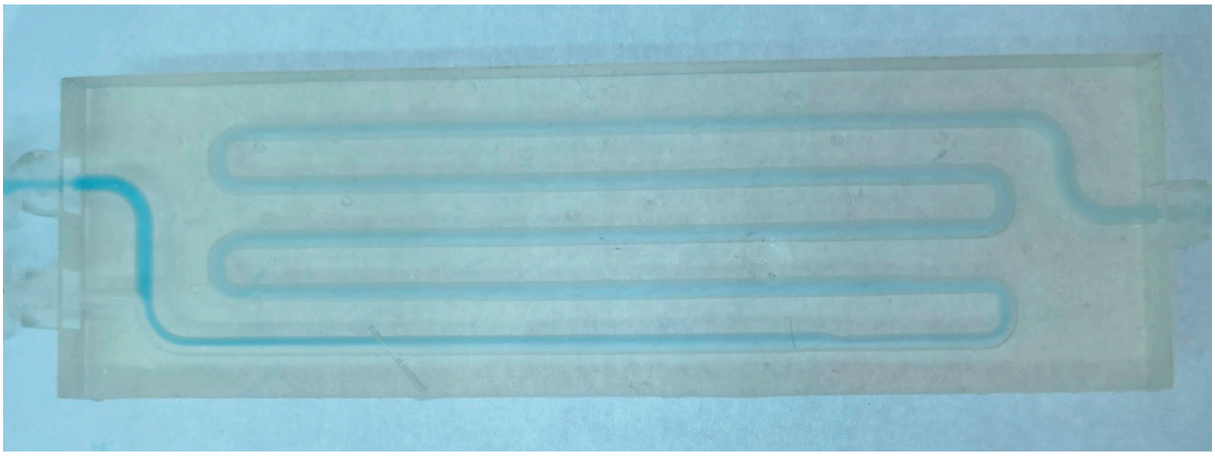

Supplementary Figure S13. Tubular millireactor at flow of 49 μL min^{-1}

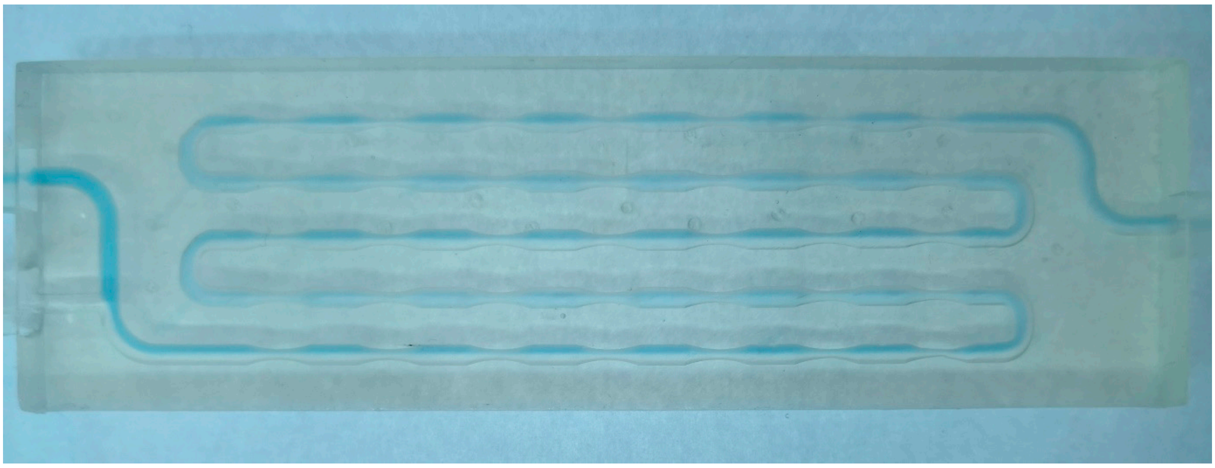

Supplementary Figure S14. Venturi millireactor at flow of 448 μL min^{-1}

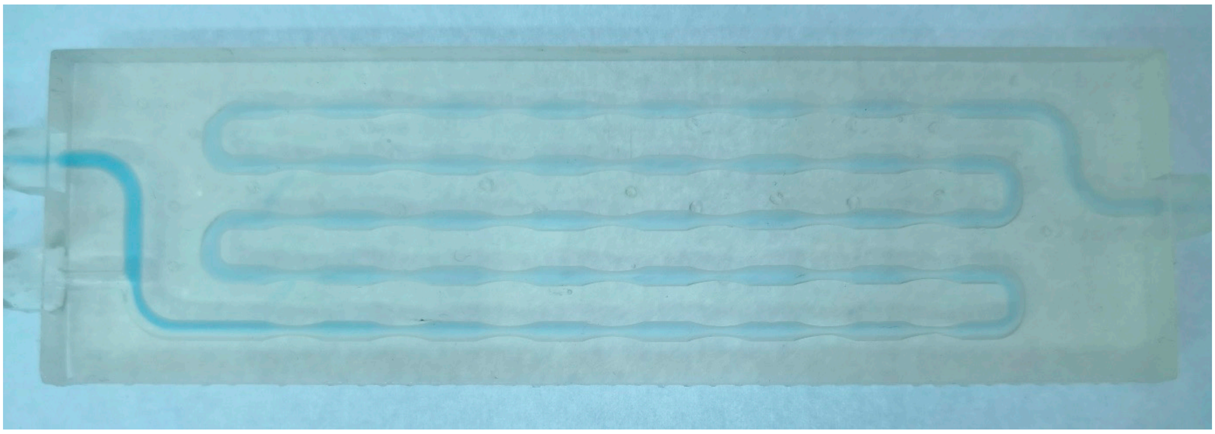

Supplementary Figure S15. Venturi millireactor at flow of 37 μL min^{-1}

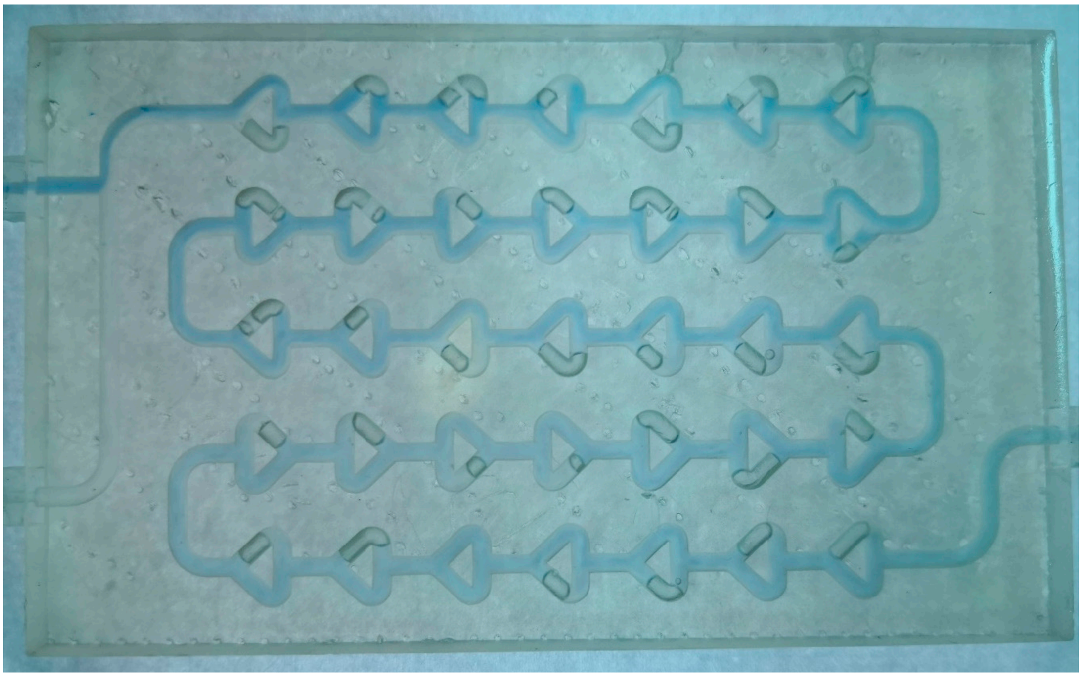

Supplementary Figure S16. Chaos millireactor at flow of 1059 μL min^{-1}

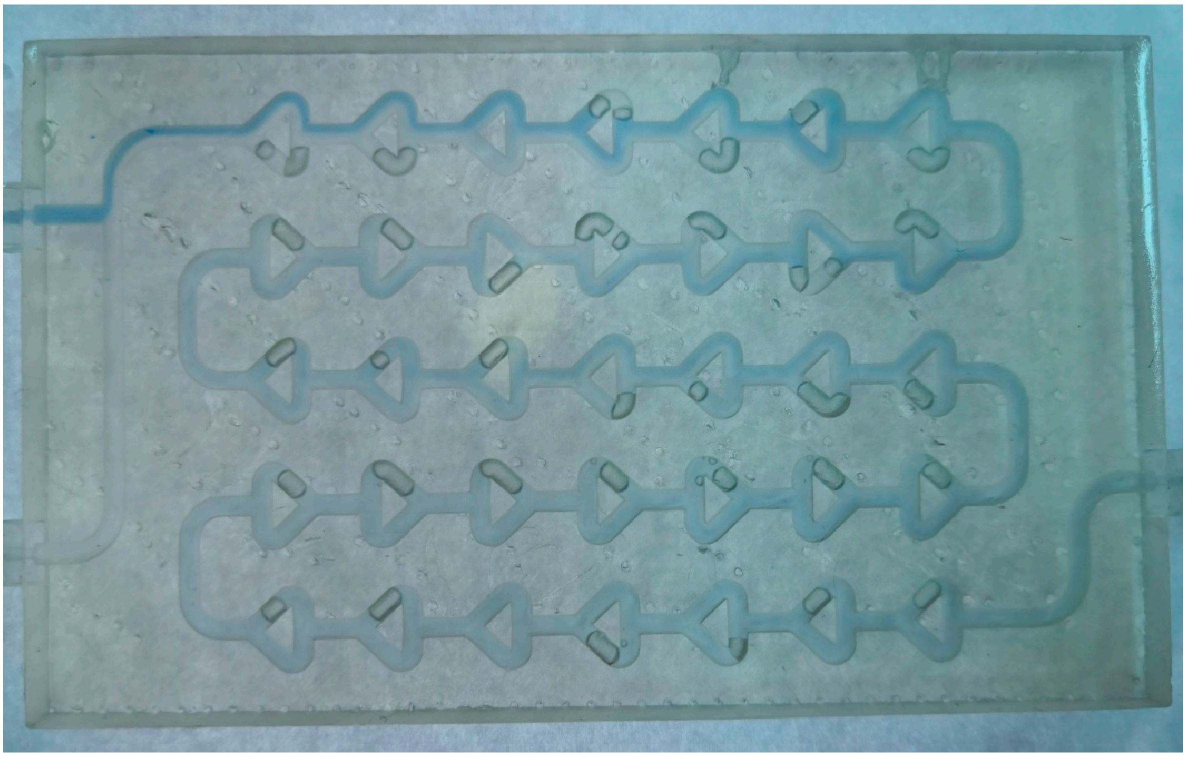

Supplementary Figure S17. Chaos millireactor at flow of 88 µL min^{-1}

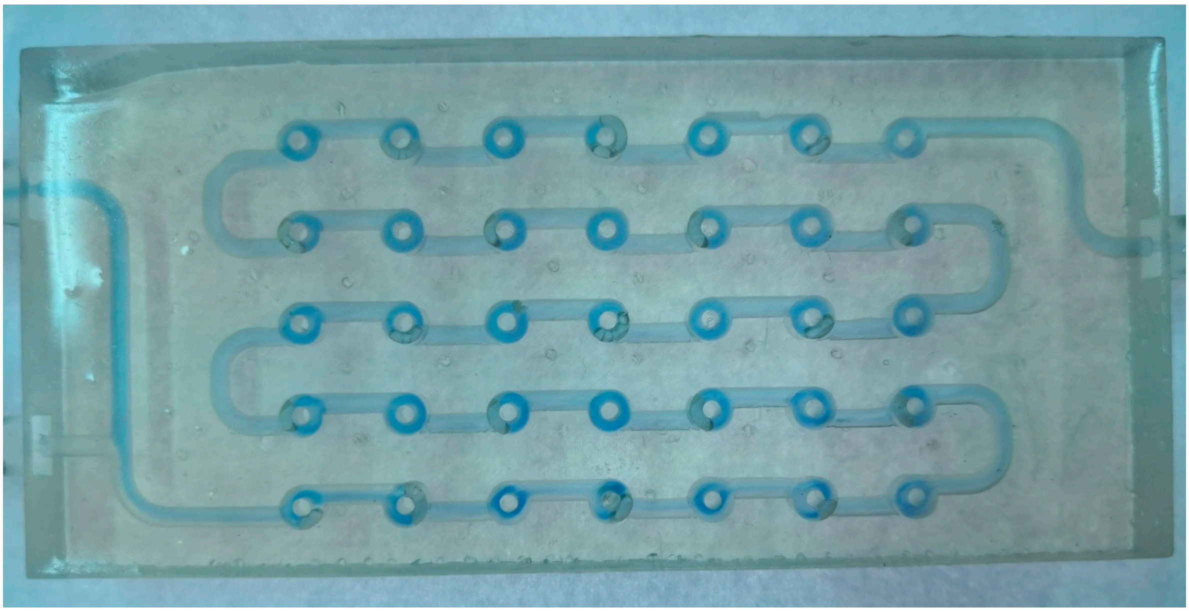

Supplementary Figure S18. Cyclone millireactor at flow of 1312 µL min^{-1}

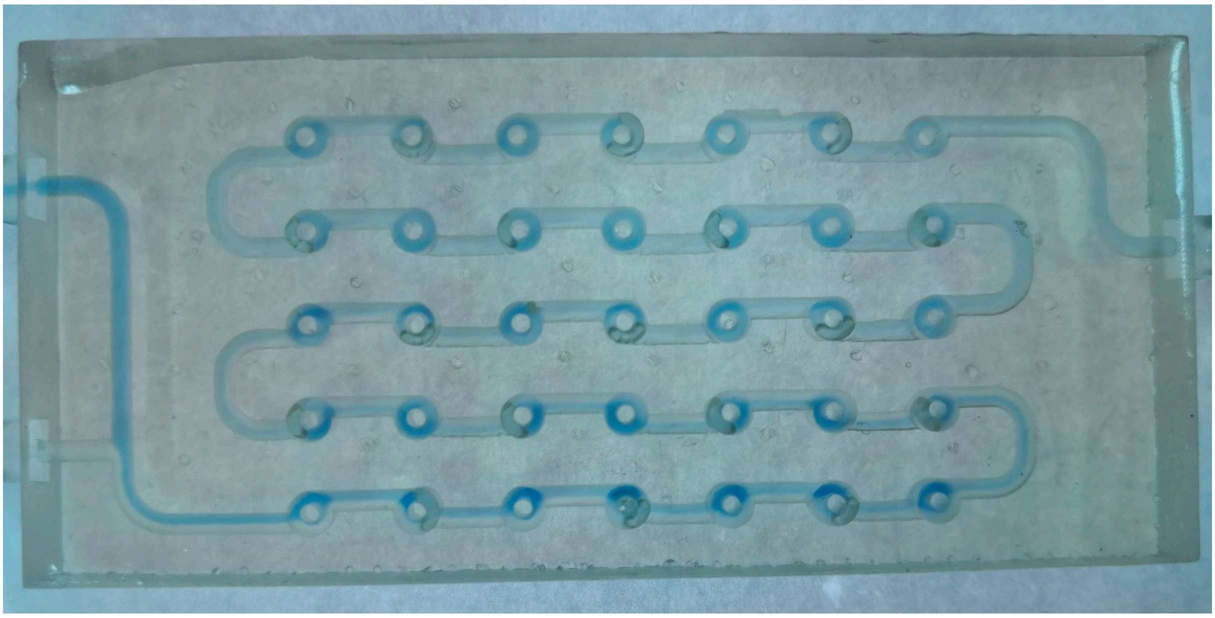

Supplementary Figure S19. Cyclone millireactor at flow of 109 μL min^{-1}

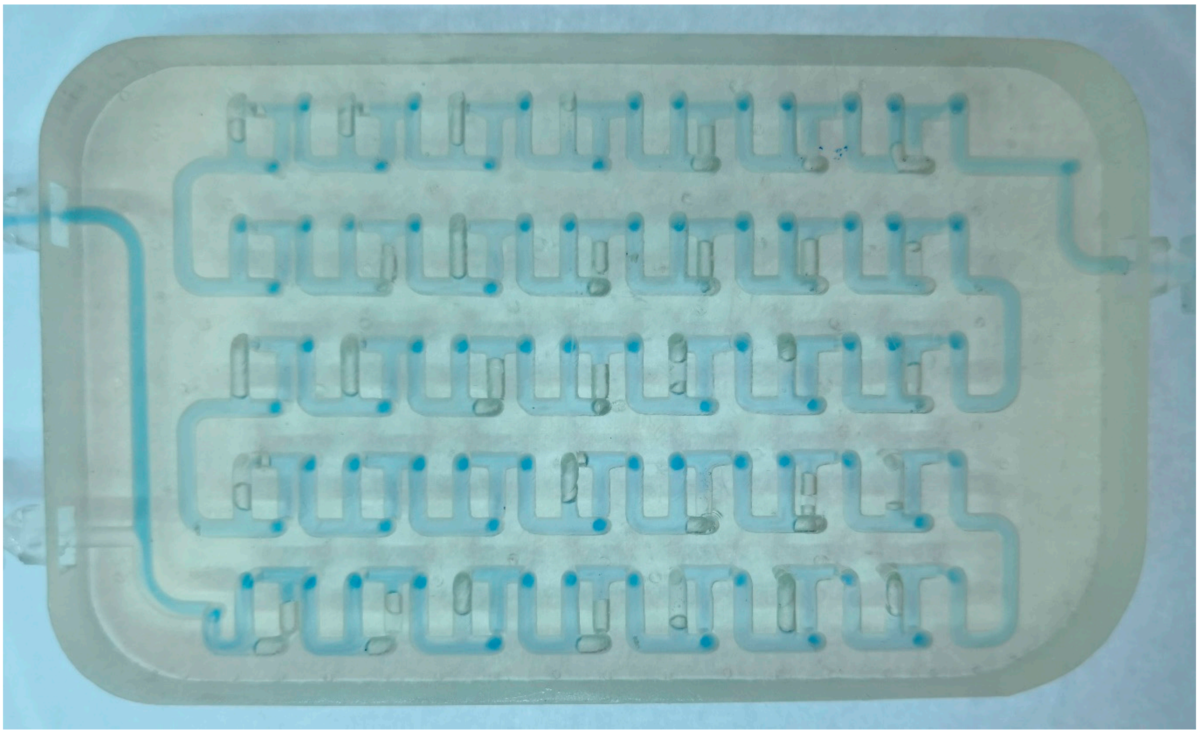

Supplementary Figure S20. Mobius millireactor at flow of 1823 μL min^{-1}

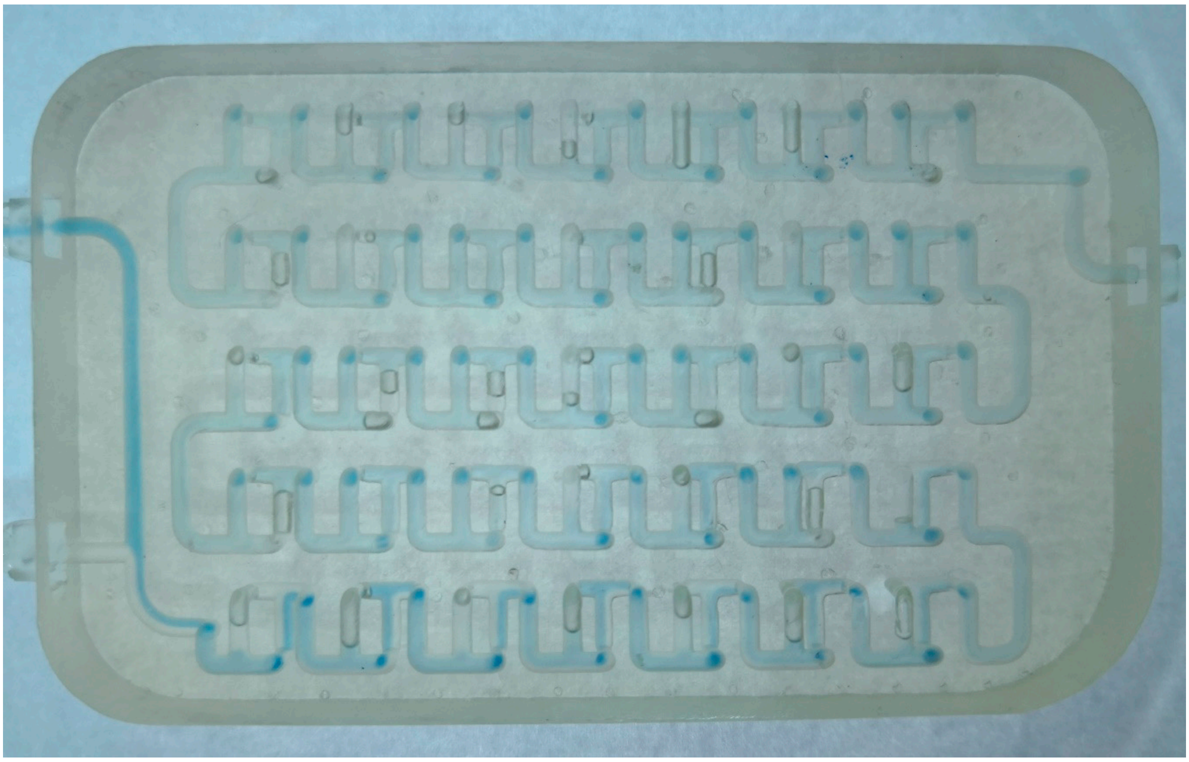

Supplementary Figure S21. Mobius millireactor at flow of 152 μL min^{-1}

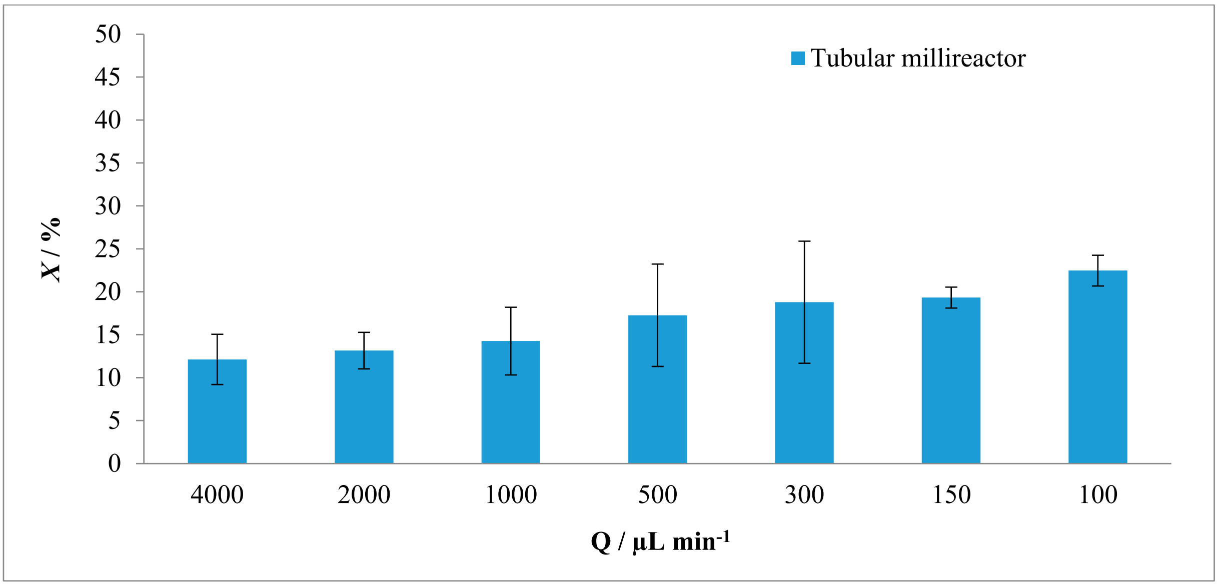

Supplementary Figure S22. Graphical representation of the conversion values obtained for the Tubular millireactor with error bars

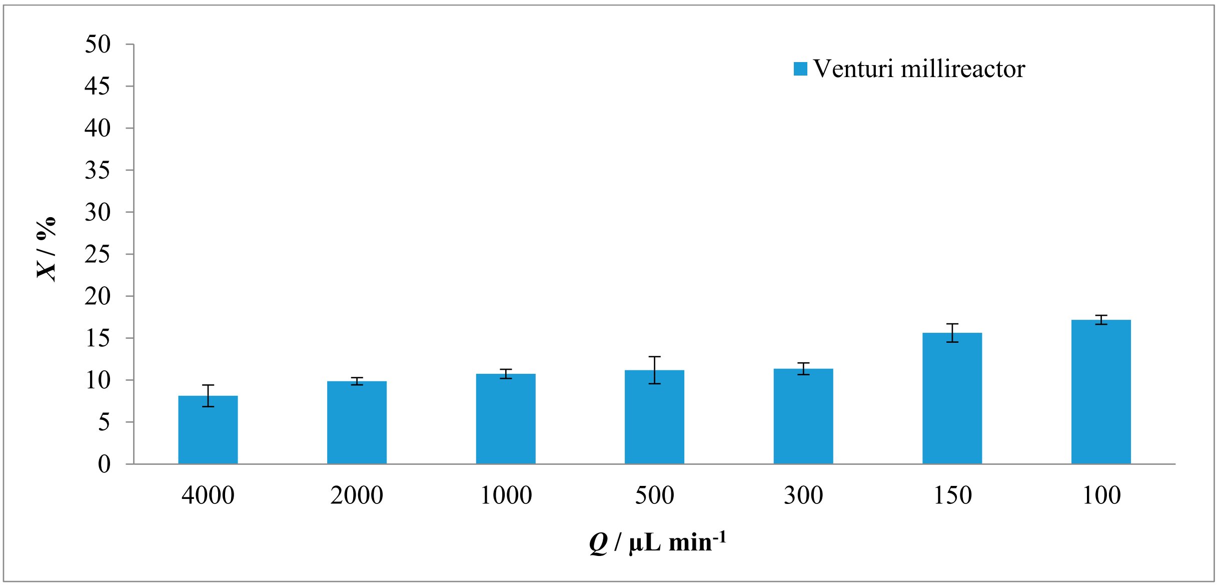

Supplementary Figure S23. Graphical representation of the conversion values obtained for the Venturi millireactor with error bars

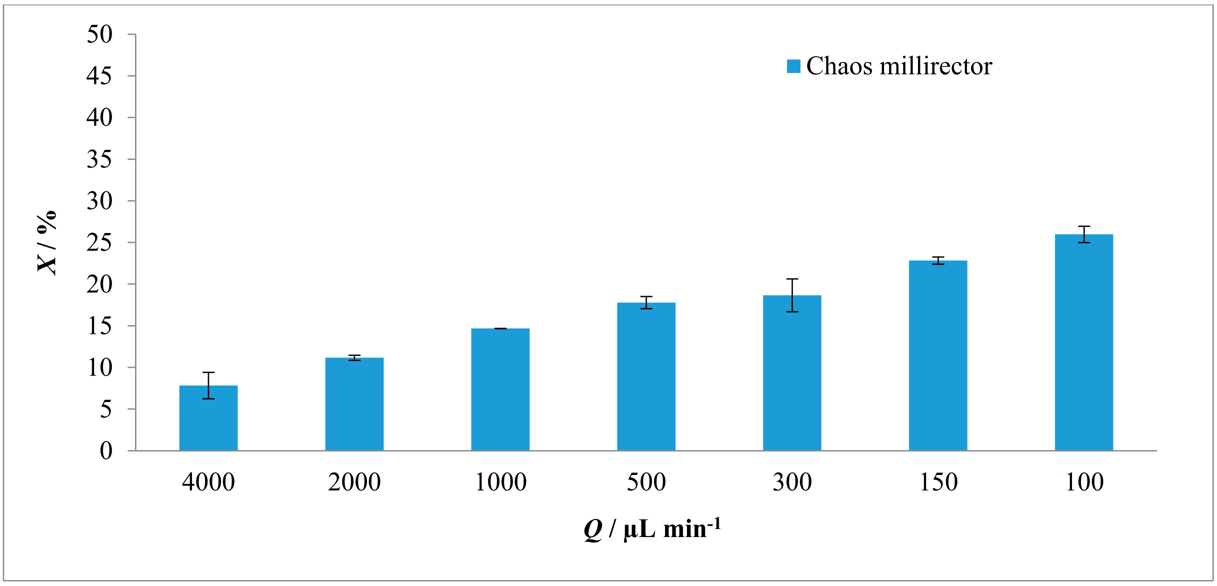

Supplementary Figure S24. Graphical representation of the conversion values obtained for the Chaos millireactor with error bars

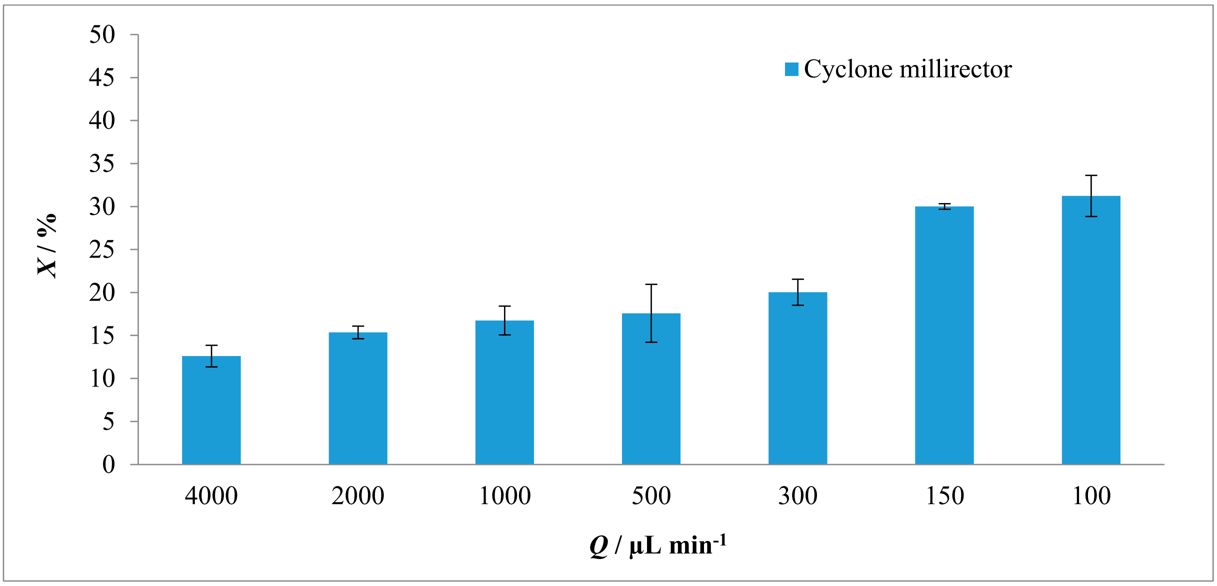

Supplementary Figure S25. Graphical representation of the conversion values obtained for the Cyclone millireactor with error bars

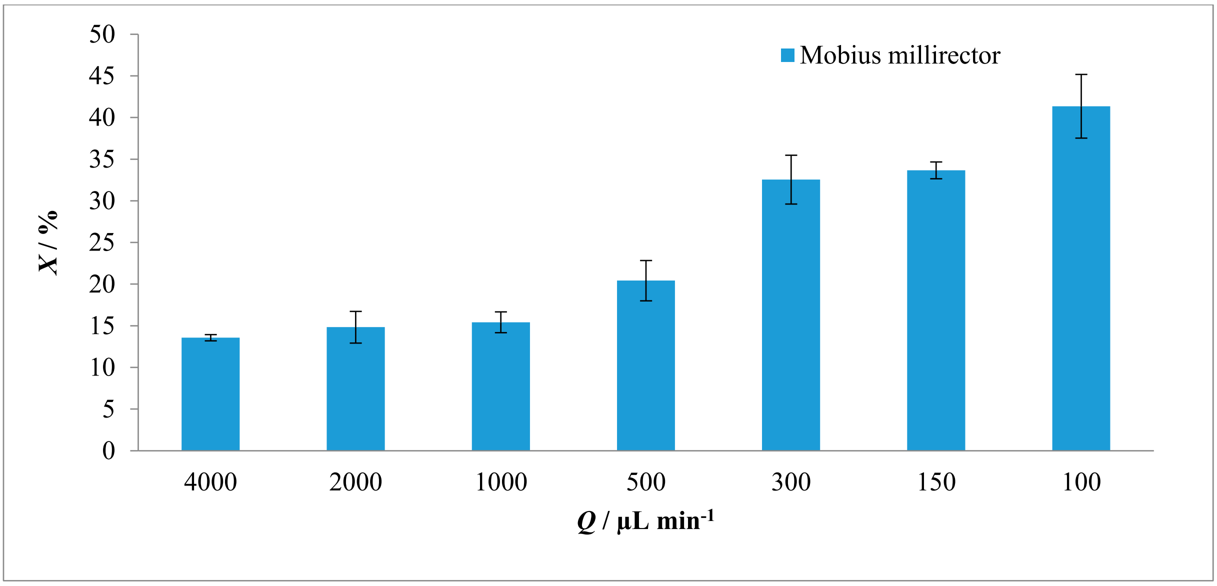

Supplementary Figure S26. Graphical representation of the conversion values obtained for the Mobius millireactor with error bars

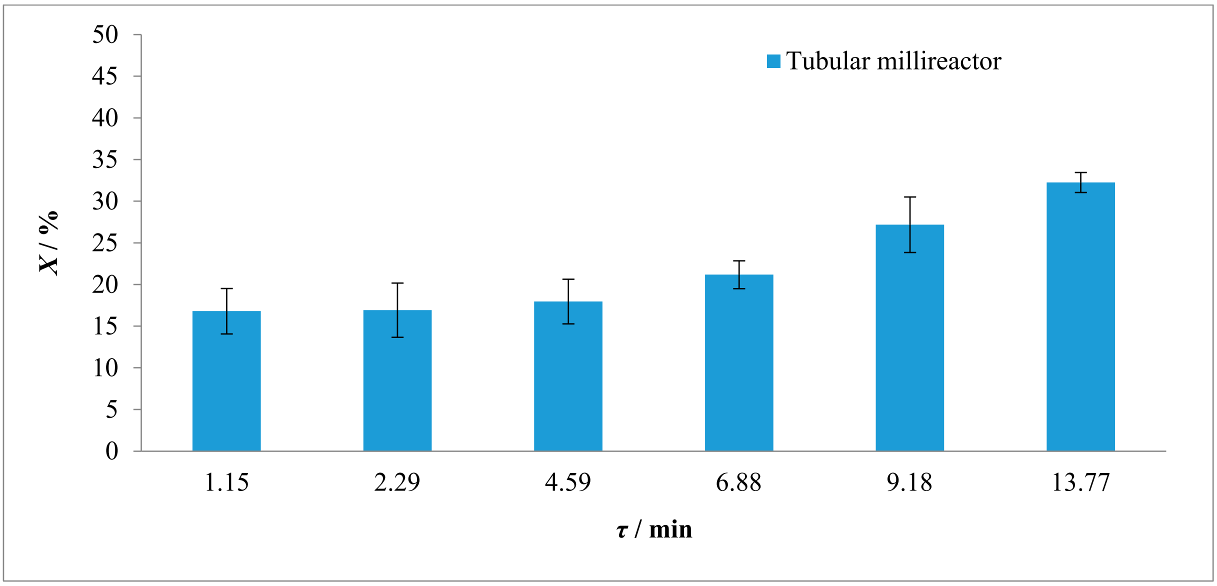

Supplementary Figure S27. Graphical representation of the dependence of the conversions of the Tubular millireactor on fixed residence times with error bars

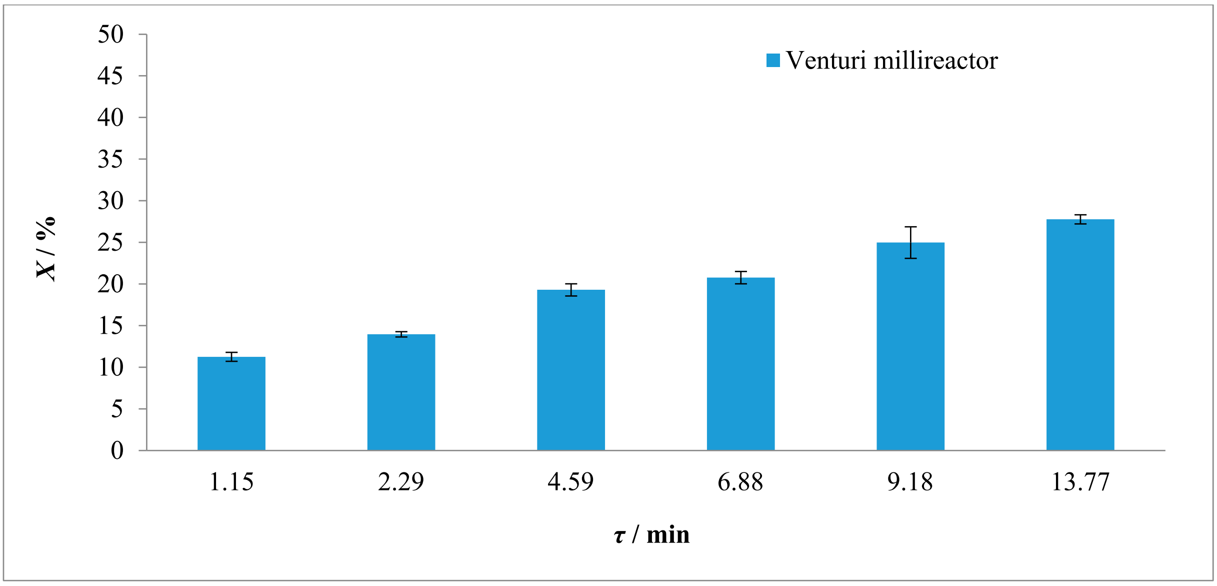

Supplementary Figure S28. Graphical representation of the dependence of the conversions of the Venturi millireactor on fixed residence times with error bars

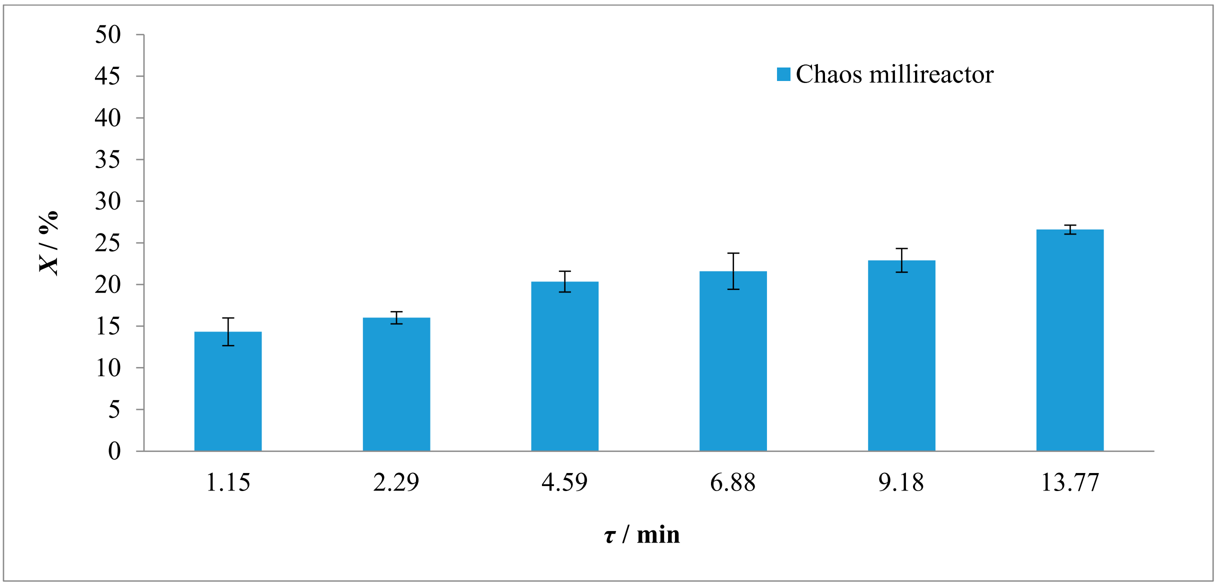

Supplementary Figure S29. Graphical representation of the dependence of the conversions of the Chaos millireactor on fixed residence times with error bars

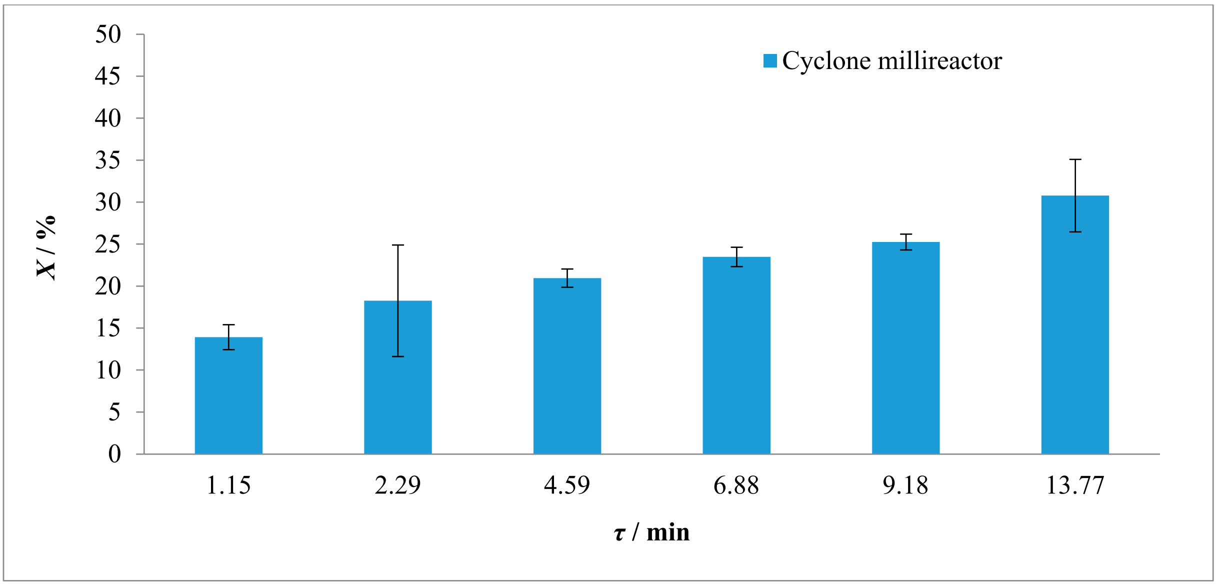

Supplementary Figure S30. Graphical representation of the dependence of the conversions of the Cyclone millireactor on fixed residence times with error bars

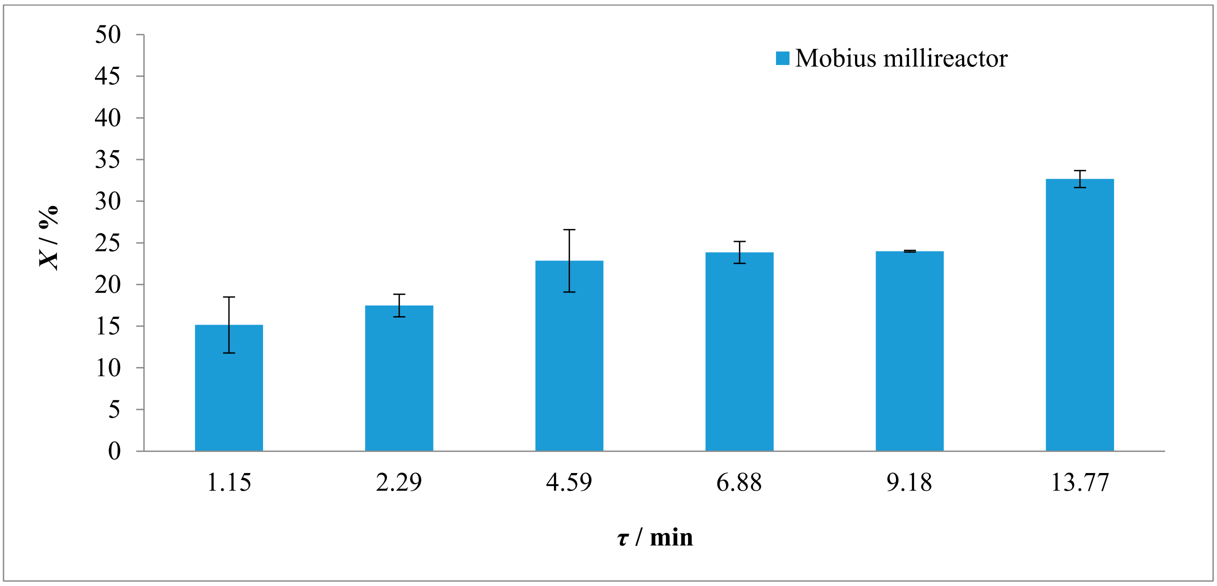

Supplementary Figure S31. Graphical representation of the dependence of the conversions of the Mobius millireactor on fixed residence times with error bars

Fenton process

The use of Fenton oxidation processes began in the late 1960s to remove various organic contaminants in water. The Fenton oxidation process is a homogeneous catalytic method that uses a mixture of iron ions (Fe^{2+}) and hydrogen peroxide (H_2O_2) in an acidic medium. The main advantages of this process are high efficiency, non-selectivity of hydroxyl radicals and the possibility of complete decomposition of pollutants into harmless compounds. The mechanism describing the Fenton process is shown in the following reactions:

$$Fe^{2+} + H_2O_2 \rightarrow Fe^{3+} + \cdot OH + OH^- \qquad (S.1)$$

$$Fe^{3+} + H_2O_2 \rightarrow Fe^{2+} + \cdot OOH + H^+ \qquad (S.2)$$

$$Fe^{2+} + \cdot OH \rightarrow Fe^{3+} + OH^- \qquad (S.3)$$

$$H_2O_2 + \cdot OH \rightarrow \cdot OOH + H_2O \qquad (S.4)$$

$$Fe^{2+} + \cdot OOH \rightarrow Fe^{3+} + HO_2^- \qquad (S.5)$$

$$Fe^{3+} + \cdot OOH \rightarrow Fe^{2+} + H^+ + O_2 \qquad (S.6)$$

Equation S.1 shows the catalytic decomposition of hydrogen peroxide by iron(II) ions into hydroxyl radicals, which are strong oxidizing agents, and iron(II) ions are oxidized to iron(III)

ions. The Fenton process can play a dual role - oxidation and coagulation in the water treatment process, since iron(II) ions and iron(III) ions are coagulants. Equation S.2 shows the regeneration of iron(II) ions, which is also the slowest reaction that determines the reaction rate. Equations S.3 and S.4 show competing reactions that also occur and negatively affect the oxidation process. In these equations, iron(II)ions and hydrogen peroxide act as scavengers of hydroxyl radicals.

The spectrum of dye RB182

An aqueous solution of dye RB182 was prepared (γ = 15 mg L^{-1}), and the dye spectrum was recorded from 800 nm to 390 nm at a rate of 200 nm min^{-1}. The highest absorbance was achieved at a wavelength of 610 nm, as shown in Supplementary Figure S5. All samples were monitored spectrophotometrically at a maximum wavelength of 610 nm.

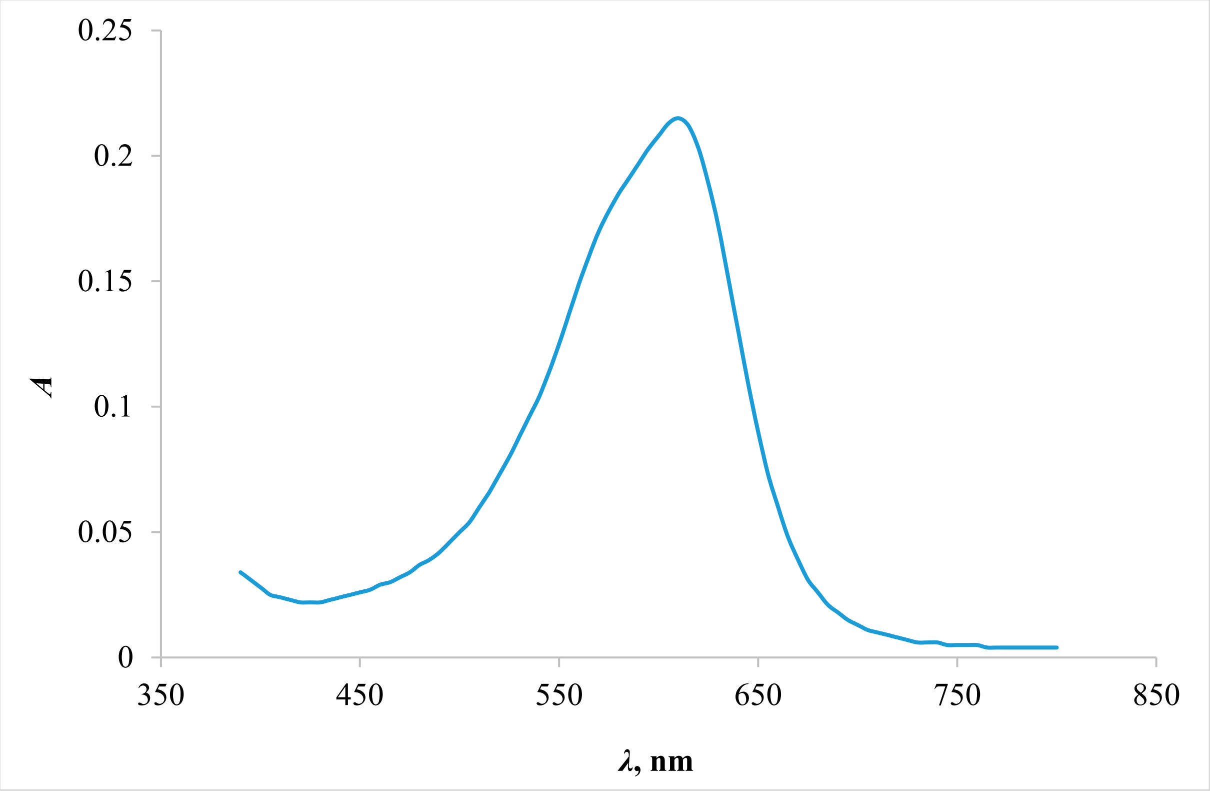

Supplementary Figure S32. Graphical representation of RB182 dye absorbance-wavelength dependence
